# Reactive astrocytes mediate toxicity in iPSC derived dopaminergic neurons

**DOI:** 10.1038/s41531-026-01378-9

**Published:** 2026-06-12

**Authors:** Naroa Ibarra-Aizpurua, Jon Olano-Bringas, Benjamin Vallin, Lucy A. Crompton, Sally A. Cowley, Nora Bengoa-Vergniory, Richard Wade-Martins

**Affiliations:** 1 Oxford Parkinson’s Disease Centre and Department of Physiology, Anatomy and Genetics, University of Oxford, Oxford, UK; 2 Kavli Institute for Neuroscience Discovery, University of Oxford, Oxford, UK; 3 Achucarro Basque Center for Neuroscience, Leioa, Spain; 4 Department of Neuroscience, University of the Basque Country (UPV/EHU), Leioa, Spain; 5 Centre for Biomedical Research, School of Applied Sciences, University of the West of England, Bristol, UK; 6 James and Lillian Martin Centre for Stem Cell Research, Sir William Dunn School of Pathology, University of Oxford, Oxford, UK; 7 Ikerbasque – Basque Foundation for Science, Bilbao, Spain

**Keywords:** Cell biology, Neurology, Neuroscience

## Abstract

Neuroinflammation is a hallmark of Parkinson’s disease (PD), a progressive neurodegenerative disorder characterized by the accumulation of α-synuclein and the death of dopaminergic neurons in the substantia nigra. Mutations in *GBA* are a common risk factor for PD, which can lead to lipid metabolism dysfunction, autophagy/lysosomal dysregulation, as well as the disruption of other cellular functions. In this study, we investigated the impact of the *GBA*-*N370S* mutation and astrocytic reactivity on α-synuclein pathology and neurotoxicity. To investigate the impact of reactive astrocytes on Parkinson’s disease pathology, we employed iPSC-derived midbrain astrocyte and dopaminergic neuron co-cultures from control and *GBA-N370S* donors, as well as primary mouse midbrain astrocyte cultures and transcriptomic assays to examine the response of astrocytes to Tumor Necrosis Factor-α (TNFα) and Interferon-γ (IFNγ). We show that upon inflammatory stimuli astrocytes become reactive, leading to extensive transcriptional changes. RNAseq and experimental validation revealed that calcium transport and homeostasis were severely dysregulated, and functional studies confirmed that *GBA-N370S* astrocytes exhibited increased calcium release when treated with cytokines. We further explored the impact of inflammation on astrocytic neurosupport in an iPSC-derived dopaminergic neuron and astrocyte co-culture model finding that combined treatment of TNFα, IFNγ and α-synuclein pre-formed fibrils (PFFs) led to neurotoxic effects, suggesting that TNFα and IFNγ-activated astrocytes mediate α-synuclein PFF toxicity. Taken together, these data provide evidence of reduced neurosupport in both control and *GBA-N370S* iPSC-derived midbrain astrocytes exposed to inflammatory cytokines, suggesting a role for reactive astrocytes in PD pathology.

## Introduction

Parkinson’s disease (PD) is a progressive neurodegenerative movement disorder characterized by the presence of motor (bradykinesia, tremor, rigidity, and postural instability) and non-motor (cognitive decline, depression, sleep disturbances, hyposmia, and gastrointestinal issues) symptoms^1,2^. Histologically, PD is characterized by the preferential loss of dopaminergic neurons (DAns) in the substantia nigra pars compacta (SNpc) of the midbrain, amongst other features^3^. Another major histopathological hallmark of the disease is the presence of proteinaceous deposits in the form of Lewy bodies (LB) composed mainly of α-synuclein^4^ and other cellular components, such as lipids and organelles^5^. Idiopathic PD accounts for 90–95% of cases, while 5–10% are associated with familial disease^6^. The *N370S* mutation in the *GBA1* gene is one of the most common genetic risk factors for PD^7,8^. *GBA1* encodes the lysosomal enzyme β-glucocerebrosidase (GCase), which catalyzes the final reaction in glycosphingolipid and ganglioside metabolism. The *GBA-N370S* mutation leads to a reduction in GCase enzymatic activity, resulting in the accumulation of its substrates^9^. Glucosylceramide, one of the main substrates for GCase, has been reported to interact with α-synuclein to induce aggregation-prone conformational changes^10^.

Various human, animal, and cell models carrying *GBA1* variants have shown a decrease in GCase activity, providing a direct link between *GBA1* mutations and α-synuclein pathology^11–13^. Indeed, studies have shown that pharmacological inhibition of GCase with conduritol-β-epoxide (CBE) results in the accumulation of α-synuclein aggregates^14^. Furthermore, various pathological mechanisms associated with *GBA1* mutations have been reported: endoplasmic reticulum (ER) stress induced by GCase retention in the ER^15^; increased oxidative stress; decreased ATP levels and mitochondrial membrane potential^16^; dysregulated calcium homeostasis^17^; and alterations in lipid and glucosylceramide and glucosylsphingosine levels^18,19^.

For many years, research into the pathogenesis of PD has focused on DAns. However, these cells do not live in isolation in the central nervous system (CNS) as they are surrounded by astrocytes, amongst other glial cells^20^. Astrocytes exhibit several functions related to neuronal support and homeostasis that are dysregulated in pathological contexts like PD: increased blood-brain barrier (BBB) permeability^21^, impaired astrocytic glutamate uptake^22^, impaired α-synuclein uptake and degradation^23^, increased inflammatory response^24^, and neurotoxicity to DAn^25^. Furthermore, many genes related to genetic PD (*GBA1*, *PARK7*, *PINK1*) are also expressed in astrocytes, leading to cell autonomous and non-cell autonomous dysfunctions^26–28^.

The inaccessibility of the CNS makes it difficult to study the pathological mechanisms that undergo in neurodegenerative diseases such as PD. Histological studies on post-mortem human brain tissue only show the very late stages of the disease, while animal models are not always translatable to humans^29^. In contrast, human induced pluripotent stem cells (iPSCs) are a useful tool for studying cellular and intercellular mechanisms in any type of cell that carries the genetic architecture of the donor, allowing us to study the pathological mechanisms involved in specific PD-related mutations such as *LRRK2-G2019S* or *GBA-N370S*^27,30–33^. In addition, protocols have been developed to generate region-specific cells, such as cortical or mesencephalic astrocytes, acknowledging that glial cells exhibit profound regional and sub-regional differences^34–36^. However, little is known about the contribution of region-specific astrocytes carrying the *GBA-N370S* mutation to the pathogenesis of PD^37^.

In this study, we describe the generation and characterization of fully mature and functional iPSC-derived midbrain astrocytes (iASTROs) from control and *GBA-N370S* donors. We demonstrate that our model is a bona fide human astrocyte model when compared to other human and iPSC-derived models, and that the iASTROs undergo profound transcriptional and functional changes in response to different inflammatory exposures. Induced dopaminergic neuron (iDAn)-iASTRO co-cultures treated with inflammatory cytokines and α-synuclein pre-formed fibrils (PFFs) exhibited neurodegeneration and astrocytic reactivity. This phenotype was recapitulated in iDAn-midbrain mouse astrocyte co-cultures, further demonstrating that astrocytes are key players in the crosstalk between α-synuclein, inflammation and neuronal survival in PD.

## Results

### Control and *GBA-N370S* iASTROs express mature astrocytic markers and present functional maturity

Astrocytic induction of human iPSCs (five control and five *GBA-N370S* lines) was performed as described in the Materials and Methods section. Induced cells were analyzed for the expression of astrocytic (S100β) and DAn (FOXA2 and TH) markers at days in vitro 30 (DIV30), 60 (DIV60), and 100 (DIV100) (Supplementary Fig. 2A, B). Most of the induced cells expressed S100β at DIV100, whereas TH expression was virtually absent. In contrast, markers of DAns were high at DIV30, and their expression levels decreased as the differentiation progressed (Supplementary Fig. 2A, B). mRNA expression levels of several astrocytic (*GFAP*, *S100B*, *CD44*), and proliferative (*MKI67*), stem cell (*SOX2*) and DAn (*TH*) markers were also measured, which increased and decreased over time, respectively, indicating that the induced cells were molecularly astroglial cells (Supplementary Fig. 3A–F). In addition, DIV100+ iASTROs were ~80% positive for S100B and ~20% positive for GFAP, which is in keeping with the literature (Supplementary Fig. 3G). These results demonstrate the generation of iPSC-derived astrocytes (iASTROs) from the different human iPSC lines used in this study.

Having shown that the induced cells expressed molecular signatures of astrocytes, we decided to investigate whether iASTROs were functional. In terms of glutamate uptake, iASTROs showed a statistically significant higher percentage of glutamate uptake when compared to iASTROs treated with DL-TBOA, an excitatory amino acid transporter (EAAT) blocker, regardless of the genotype (Supplementary Fig. 3H).

Taken together, these results demonstrate that our model successfully generates mature functional iPSC-derived astrocytes, as revealed by their molecular characterization.

### Control and *GBA-N370S* iASTROs support the health and survival of control and *GBA-N370S* iDAns

Astrocytes play a key role in the homeostasis and survival of neurons. To determine whether the generated iASTROs support the health and survival of iPSC-derived DAns, DIV100 control and *GBA-N370S* iASTROs were co-cultured with iPSC-derived control and *GBA-N370S* dopaminergic neurons (iDAns) for 30 days.

**Fig. 1:**
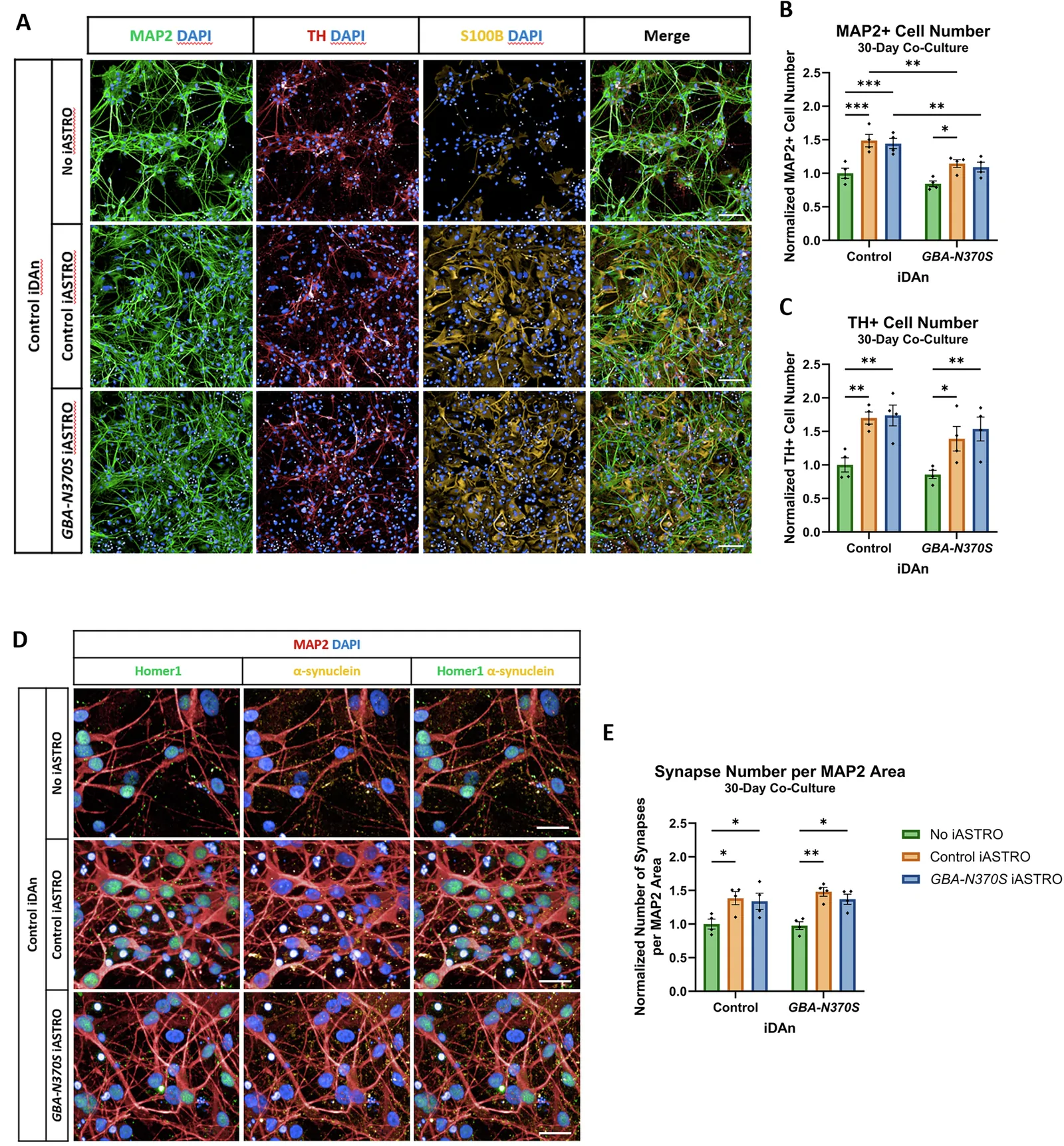
Generating *GBA-N370S* and control iDAn-iASTRO co-cultures. **A** Representative immunocytochemistry images of control iDAn at DIV45, either in monoculture or after 10 days of co-culture with control or *GBA-N370S* iASTROs, stained for MAP2 (green), TH (red), S100B (yellow) and DAPI nuclear staining (blue). Scale bar: 100 μm. **B**, **C** Quantifications of MAP2+ and TH+ cell number in iDAn monoculture or co-culture with control or *GBA-N370S* iASTROs for 30 days. Each data point represents 1 iDAn line, averaged in each differentiation to all the conditions either in monoculture or co-cultured with 2 lines of iASTROs of each genotype, and then averaged across differentiations. For each differentiation, values were normalized to the average of control iDAn in monoculture. *N* = 3 control and 3 *GBA-N370S* lines for iDAn (3 differentiations), and *N* = 3 control and 3 *GBA-N370S* lines for iASTROs (1–3 differentiations). Mean ± SEM. The statistical analysis performed was a two-way ANOVA with Tukey’s multiple comparison test. *p < 0.05, **p < 0.01, ***p < 0.001. **D** Representative immunocytochemistry images of control iDAn in monoculture or in co-culture for 30 days with control or *GBA-N370S* iASTROs, strained for MAP2 (red), Homer1 (green), α-synuclein (yellow) and DAPI nuclear staining (blue). Scale bar: 25 μm. **E** Quantification of the number of Homer1 and α-synuclein co-localized spot number per MAP2 area, indicating synaptic apposition, in control and *GBA-N370S* iDAn monocultures or in co-cultures with control and *GBA-N370S* iASTROs for 30 days. Each data point represents 1 iDAn line, averaged in each differentiation to all the conditions either in monoculture or co-cultured with 2 lines of iASTROs of each genotype, and then averaged across differentiations. For each differentiation, values were normalized to the average of control iDAn in monoculture. *N* = 3 control and 3 *GBA-N370S* lines for iDAn (3 differentiations), and *N* = 3 control and 3 *GBA-N370S* lines for iASTROs (1–3 differentiations). Mean ± SEM. The statistical analysis performed was two-way ANOVA with Tukey’s multiple comparison test. *p < 0.05, **p < 0.01, ***p < 0.001, ****p < 0.0001.

The presence of either control or *GBA-N370S* iASTROs significantly increased the number of MAP2-positive (MAP2+) neurons compared to iDAn monocultures (Fig. 1A, B). We observed a small reduction in the number of MAP2+ neurons in co-cultures with *GBA-N370S* iDAns compared to the co-culture conditions with control iDAns (Fig. 1B). Similar results were obtained when quantifying the number of TH-positive iDAns, with a significant increase in the number of TH-positive cells in the presence of both control and *GBA-N370S* iASTROs compared to the monocultures (Fig. 1C). Interestingly, we observed a trend towards a lower number of TH-positive (TH+) cells in co-cultures with *GBA-N370S* iDAns compared to those with control iDAns (Fig. 1C).

Consistent with these results, a significant increase in synapse formation was observed in the presence of either control or *GBA-N370S* iASTROs at DIV30 compared to iDAn monocultures, thus suggesting that iASTROs support synapse development and this function does not appear to be affected by the *GBA-N370S* mutation (Fig. 1D–E). Collectively, these results show that both control and *GBA-N370S* iASTROs support neuronal survival in co-culture with iDAns, with no differences due to the iASTRO genotype, although *GBA-N370S* iDAns appear to be more vulnerable than control iDAns.

### RNAseq validates iASTROs as a bona fide human astrocyte model which responds to proinflammatory stimuli

**Fig. 2:**
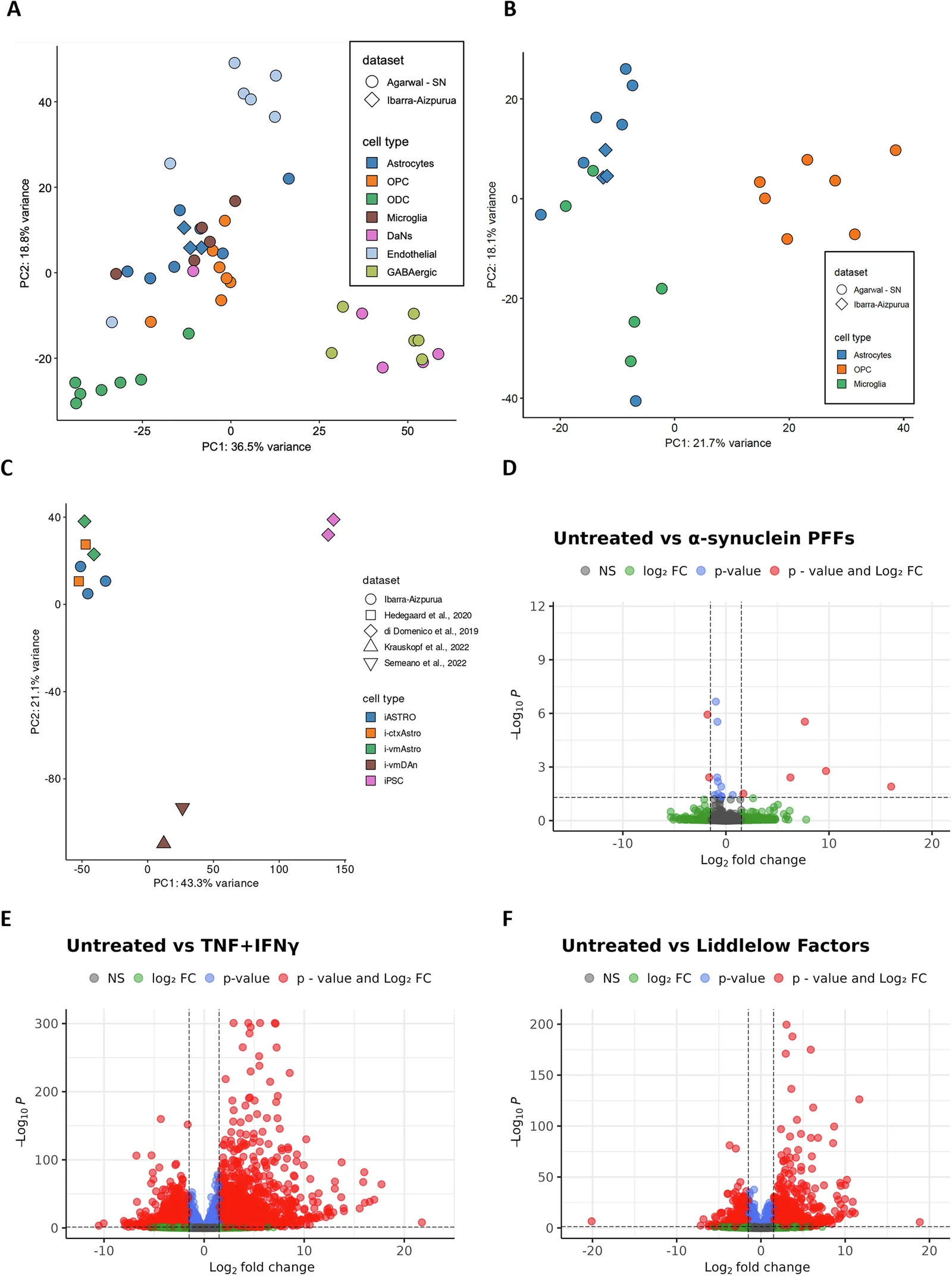
RNAseq validates iASTROs as a bona fide human astrocyte model which responds to proinflammatory stimuli. **A**, **B** PCA plot of (left) bulk RNAseq data of 3 control iASTROs and scRNAseq data of different human SN cell types and (right) of astrocyte, oligodendrocyte precursor cell (OPC) and microglia cell types. For **A**, the scRNAseq dataset was obtained from the Agarwal et al.^38^ paper, accession code GSE140231. **C** PCA plot of bulk RNAseq data of 3 control iASTROs, iPSC-derived midbrain astrocytes (i-vmAtro-1/2), iPSC-derived cortical astrocytes (i-ctxAtro-1/2), iPSC-derived midbrain DAn (i-vmDAn-1/2), and iPSCs. **D**–**F** Volcano plots showing the DEGs in control iASTROs treated with α-synuclein PFFs, TNF + IFNγ, and TNF + IL-1α + C1q compared to untreated control iASTROs. Each dot represents one gene. Gray dots represent not differentially expressed genes; green dots represent genes with a Log2FoldChange <−1.5 or >1.5 but a *p*_adj_>0.05, blue dots represent genes with a *p*_adj_<0.05 but a Log2FoldChange >−1.5 and <1.5; red dots represent genes with a Log2FoldChange <−1.5 or >1.5 and *p*_adj_<0.05. All treatment concentrations are as follows: 30 ng/mL TNF + 50 ng/mL IFNγ, the Liddelow factors (30 ng/mL TNF + 3 ng/mL IL-1α + 400 ng/mL C1q) and 10 μg/mL α-synuclein PFFs.

To determine whether our model was a bona fide human astrocyte model, we performed bulk RNA sequencing (bulk-RNAseq). We compared the bulk-RNAseq of our lines with the transcriptomic profile of a human single-cell RNAseq dataset obtained from the cortex (CX) and substantia nigra (SN) of control and PD patients^38^. Hierarchical clustering grouped iASTROs closer together with human SN astrocytes than to CX astrocytes, demonstrating their regional identity (Supplementary Fig. 4A). Furthermore, when iASTROs were compared with the different cell types of the SN using principal component analysis (PCA), iASTROs clustered closely with astrocytes, microglia, and oligodendrocyte precursor cells (OPCs) (Fig. 2A). When the most dissimilar cell populations were removed from the dataset, iASTROs clustered closely with human astrocytes in the PCA analysis (Fig. 2B). We then compared our bulk-RNAseq dataset with other bulk RNAseq transcriptomic data from iPSCs, iDAns and iPSC-derived cortical and midbrain astrocytes published in different studies (Fig. 2C). Comparisons with other bulk RNAseq datasets showed that iASTROs clustered the closest to iPSC-derived midbrain astrocytes, followed by iPSC-derived cortical astrocytes, while iPSC-derived DAns and iPSCs each formed their own clusters (Fig. 2C). Overall, these data confirm the midbrain and astrocytic genetic identity of iASTROs.

Next, we investigated the response of iASTROs to multiple inflammatory cytokines, as it is now accepted that astrocytes undergo dynamic and context-dependent cellular changes which can vary greatly depending on multiple factors, including the specific cytokines with which they are stimulated (Fig. 2D–F). To achieve this, DIV100 iASTROs were treated for 24 h with: TNF (30 ng/mL) (T); IFNγ (50 ng/mL); TNF (30 ng/mL) + IFNγ (50 ng/mL); TNF (30 ng/mL) + IL-1α (3 ng/mL) + C1q (400 ng/mL); α-synuclein pre-formed fibrils (PFFs) (10 μg/mL); or TNF (30 ng/mL) + IFNγ (50 ng/mL) + α-synuclein PFFs (10 μg/mL). An untreated control was also included. Compared to untreated iASTROs, cells treated with TNF + IFNγ showed the highest transcriptomic dysregulation with 8280 differentially expressed genes (DEGs) and 680 affected Gene Ontology (GO) terms, almost double that of the treatment groups (Fig. 2D–F and Supplementary Tables 1 and 2). Pathways related to immune and inflammatory response were activated, whereas cognition, behavior and synapse assembly-related pathways were suppressed in the TNF + IFNγ group when compared to the untreated group (Supplementary Fig. 5). In contrast, PFFs did not induce major transcriptional changes in iASTROs after 24 h of treatment (17 DEGs), being the condition with the lowest number of DEGs and GO term changes (Fig. 2D–F and Supplementary Tables 1 and 2). As Complement Component 3 (C3) upregulation and Nuclear Factor kappa-light-chain-enhancer of activated B cells (NFkB) nuclear translocation have been described to mediate inflammatory response in astrocytes, we investigated whether we could observe these effects at the protein level in order to confirm our transcriptomic observations. Indeed, iASTROS treated with TNF + IFNγ suffered C3 upregulation and NFkB nuclear translocation, the last of which was still present 7 days after treatment (Supplementary Fig. 6A–E). *C3* and *NFkB* were also upregulated at the mRNA level, and trends toward upregulation were detected for *CCL5*, *CCL2*, and *IL-6*, in agreement with the data from our RNAseq analysis (Supplementary Fig. 6F–T). Overall, these results show vast changes in gene expression in iASTROs treated with inflammatory cytokines for 24 h, especially the combined treatment of TNF and IFNγ, which we decided to further investigate in subsequent experiments.

### Widespread calcium dysregulation in reactive and *GBA-N370S* iASTROs astrocytes is recapitulated in primary mouse midbrain astrocytes

**Fig. 3:**
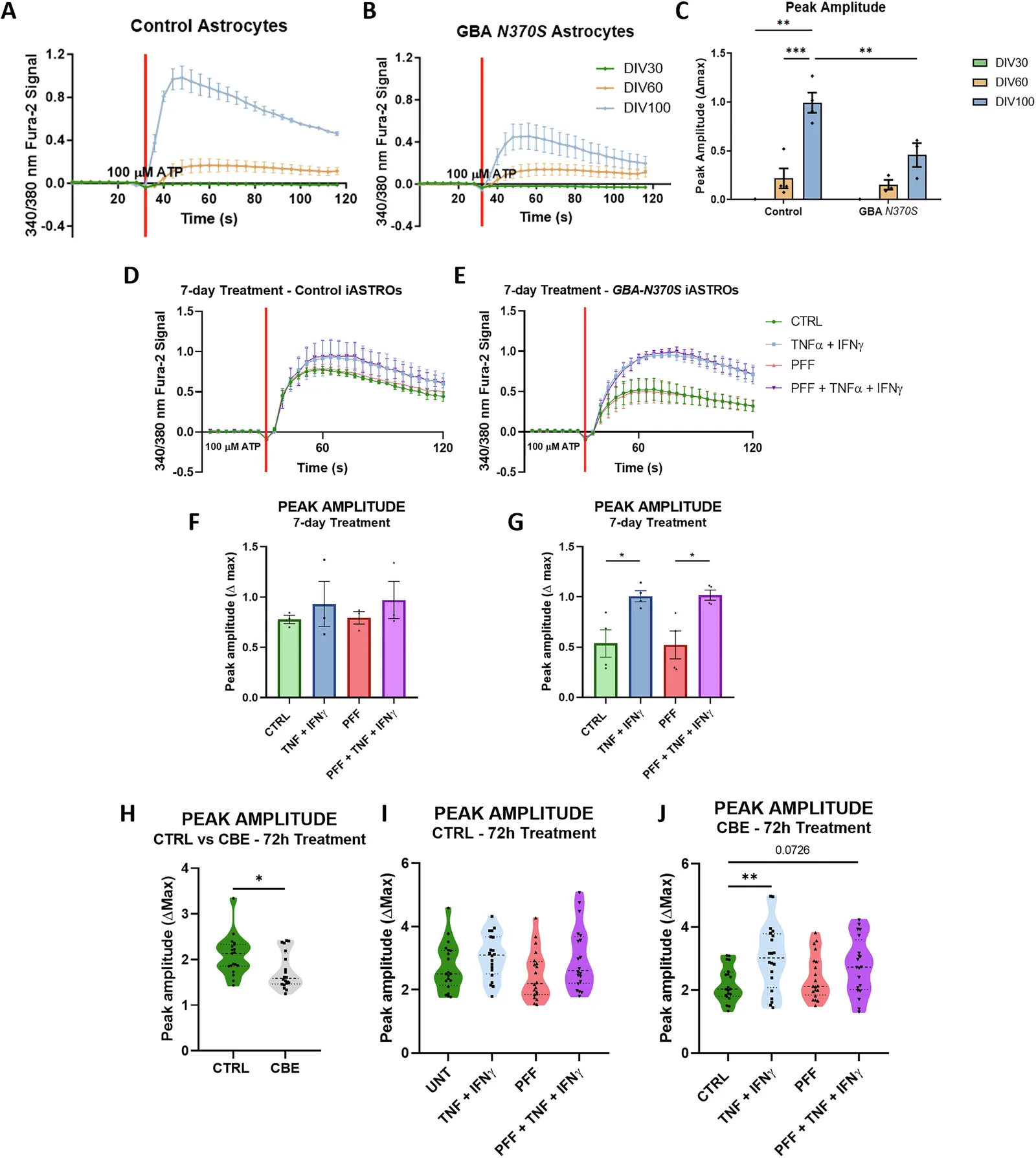
Altered calcium release is increased in *GBA-N370S* iASTROs and CBE-treated primary midbrain astrocytes treated with TNF + IFNγ and PFFs + TNF + IFNγ. **A**, **B** Traces showing the average of the Fura-2 ratio in DIV30, 60, and 100 control and *GBA-N370S* iASTROs. **C** Quantification of the peak amplitude of the traces shown in (**A**, **B**). **D**, **E** Traces showing the average of the Fura-2 ratio in control and *GBA-N370S* iASTROs treated with 30 ng/mL TNF + 50 ng/mL IFNγ, 10 μg/mL α-synuclein PFFs and 30 ng/mL TNF + 50 ng/mL IFNγ + 10 μg/mL α-synuclein PFFs. **F**, **G** Quantifications of the peak amplitude of the traces shown in (**D**, **E**). **H**–**J** Quantifications of the peak amplitude of control vs CBE-treated conditions and of control and CBE-treated astrocytes treated with 30 ng/mL TNF + 50 ng/mL IFNγ, 10 μg/mL α-synuclein PFFs and 30 ng/mL TNF + 50 ng/mL IFNγ + 10 μg/mL α-synuclein PFFs for 72 h. Each data point represents one line averaged across differentiations. *N* = 4 control and 3 *GBA-N370S* lines and N = 4 control and CBE-treated astrocytes, 1–3 differentiations. Mean ± SEM. The statistical analysis performed was two-way ANOVA with Tukey’s multiple comparison test. *p < 0.05, **p < 0.01.

Interestingly, RNAseq analysis also revealed an upregulation of calcium homeostasis and transport pathways in iASTROs treated with TNF + IFNγ (Supplementary Fig. 7A). We have previously shown that the *GBA-N370S* mutation can cause calcium dysregulation in DAns, which is known to be a critical function for both neurons and astrocytes^17^. We therefore decided to examine calcium activity in control and *GBA-N370S* iASTROs. At DIV100, *GBA-N370S* iASTROs showed lower ATP-mediated ER calcium release when compared to control iASTROs (Fig. 3A –C). Based on these findings, we decided to investigate whether control or *GBA-N370S* iASTROs exhibit dysregulation in ER-mediated calcium release upon treatment with TNF + IFNγ, α-synuclein PFFs and the combination of both, PFFs+TNF + IFNγ (Fig. 3D–G). Firstly, we confirmed that all iASTROs showed an increase in *ITPR1* and *ITPR3* following treatment with TNF + IFNγ, matching our initial RNAseq results (Supplementary Fig. 7B–C). In control iASTROs, cells treated with TNF + IFNγ for 7 days had a slightly greater increase in ER calcium release than untreated cells, but this increase was not statistically significant (Fig. 3D, F). In *GBA-N370S* iASTROs, cells treated with TNF + IFNγ for 7 days had a greater increase in calcium release than untreated cells (Fig. 3E, G). In contrast, neither control nor *GBA-N370S* iASTROs showed any differences in calcium release when stimulated with PFFs alone (Fig. 3D–G). Taken together, these results show a widespread calcium dysregulation in *GBA-N370S* iASTROs, characterized by a reduced endogenous calcium response in untreated *GBA-N370S* iASTROs which is dramatically increased by TNF + IFNγ compared to control iASTROs.

In parallel, we tested the ATP-induced ER calcium release in an orthogonal model, namely, primary midbrain astrocytes (Fig. 3H–J). This time, to mimic the *GBA-N370S* mutation, primary astrocytes were treated with 1 mM CBE. We first confirmed its effect by treating the cells with CBE for 72 h. As expected, CBE treatment reduced the peak amplitude compared to control cells (Fig. 3H). We then analyzed the calcium release from control and CBE-treated astrocytes treated with TNF + IFNγ, PFFs and PFFs+TNF + IFNγ. After 72 h post-treatment, none of the treatments induced significant differences in the calcium release from control astrocytes, compared to untreated group (Fig. 3I). However, TNF + IFNγ and PFFs+TNF + IFNγ-treated groups showed an increase in calcium release that did not reach statistical significance (Fig. 3I). In contrast, CBE-treated astrocytes exposed to TNF + IFNγ showed significantly higher calcium release compared to untreated cells, while PFFs+TNF + IFNγ treatment showed a trend for increased calcium release but did not reach statistical significance (Fig. 3J). Gene expression analysis confirmed a pro-inflammatory reaction in primary astrocytes comparable to that of iASTROs (Supplementary Fig. 7D–M). Altogether, these results show that CBE treatment for 72 h induced similar ATP-induced calcium release dysregulation in primary midbrain astrocytes compared to *GBA-N370S* iASTROs, and that this calcium release was increased upon the treatment with TNF + IFNγ and PFFs+TNF + IFNγ as previously demonstrated in iASTROs.

We next decided to investigate whether partial blockade of the ER calcium release might affect the reactivity of the iASTROs using the inositol triphosphate (ITPR) channel blocker 2-aminoethoxydiphenyl borate (2-APB)^39^ (Supplementary Fig. 8). However, no differences were observed between TNF + IFNγ and TNF + IFNγ + 2-APB treatments in any of the parameters measured, nor in the mRNA expression of inflammatory markers (CCL5, C3 and NFκB) (Supplementary Fig. 8E–K), confirming that the partial blockade of ER calcium release by 2-APB does not affect to the response to TNF + IFNγ.

Store-operated calcium entry (SOCE) is a calcium entry pathway involved in calcium homeostasis. We decided to investigate SOCE in control and *GBA-N370S* iASTROs treated with TNF + IFNγ for 24 h and 7 days (Supplementary Fig. 9). Treatment with TNF + IFNγ for 24 h did not induce significant differences in any of the genotypes when compared to untreated iASTROs, but the treatment induced a statistically significant increase in the peak amplitude of *GBA-N370S* iASTROs compared to treated control iASTROs. After 7 days, TNF + IFNγ treatment did not induce differences in control or *GBA-N370S* iASTROs when compared to untreated iASTROs, but again the peak amplitude of *GBA-N370S* iASTROs was significantly higher compared to control iASTROs. Taken together, these results show that *GBA-N370S* iASTROs have increased store-operated calcium entry (SOCE) compared to control iASTROs, whereas the treatment with TNF + IFNγ has no significant effect on SOCE in either genotype.

**Fig. 4:**
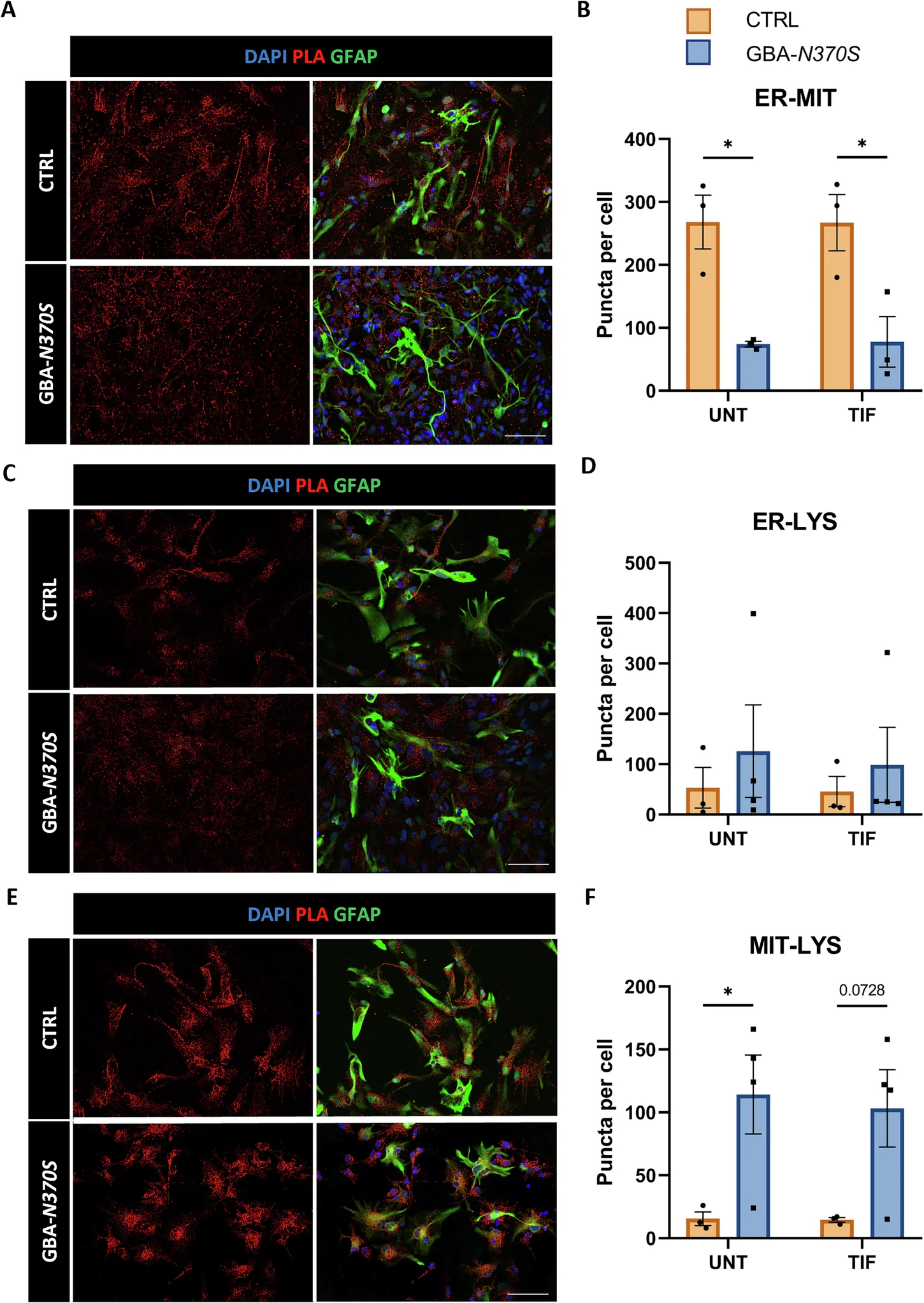
PLA reveals organelle proximity disruption in *GBA-N370S* iASTROS. Representative immunocytochemistry and PLA images of control and *GBA-N370S* iASTROs untreated or treated with 30 ng/mL TNF + 50 ng/mL IFNγ of **A** ER-mitochondria, **C** ER-lysosomes and **E** mitochondria-lysosomes interactions, stained for GFAP (green), PLA (red) and DAPI nuclear staining (blue). Scale bar of 125 µm. Quantifications of PLA puncta per cell of **B** ER-mitochondria, **D** ER-lysosomes and **F** mitochondria-lysosomes interactions. *N* = 3 control and 3-4 *GBA-N370S* lines, 1–3 differentiations. Mean ± SEM. The statistical analysis performed was unpaired t test with Welch’s correction. *p < 0.05.

Given the importance of the ER and mitochondria for ATP-induced ER calcium release, the observed widespread calcium dyshomeostasis in *GBA-N370S* iASTROs and the key role of the *GBA-N370S* mutation on lysosomal biology, we decided to investigate the interactions between these organelles by Proximity Ligation Assay (PLA) in both control and *GBA-N370S* iASTROs cultured for 7 days. To this end, we used our pre-established mit-ER (mitochondria) PLA assay^17^, and we developed two novel ER-lysosome (lys) and mit-lys-PLA assays that were able to detect the changes in proximity of these organelles in response to chloroquine treatment in SH-SY5Y cells (Supplementary Fig. 10A–G). Control iASTROs showed a significantly higher number of ER-mitochondria interactions per cell compared to *GBA-N370S* iASTROs (Fig. 4A, B). In contrast, *GBA-N370S* iASTROs showed higher mitochondria-lysosome interactions per cell compared to control iASTROs, with no significant differences observed in ER-lysosome interactions between genotypes (Fig. 4C–F). Interestingly, inter-organellar contacts were unchanged after TNF and IFNγ treatment; this suggests that genotype-specific calcium homeostasis is likely associated with perturbed inter-organellar contacts, while its stimulation under inflammatory conditions is likely driven by a different mechanism or outside of the detection range of PLA. These results firstly confirm the reduced ER-mit interactions in *GBA-N370S* iASTROs compared to control iASTROs, which we previously reported in iPSC-derived DAns^17^ and secondly, reveal the dysregulation of lysosome-mitochondria interactions in *GBA-N370S* iASTROs, which may lead to the observed calcium release perturbations.

### Reduced neurosupportive effects by reactive iASTROs

**Fig. 5:**
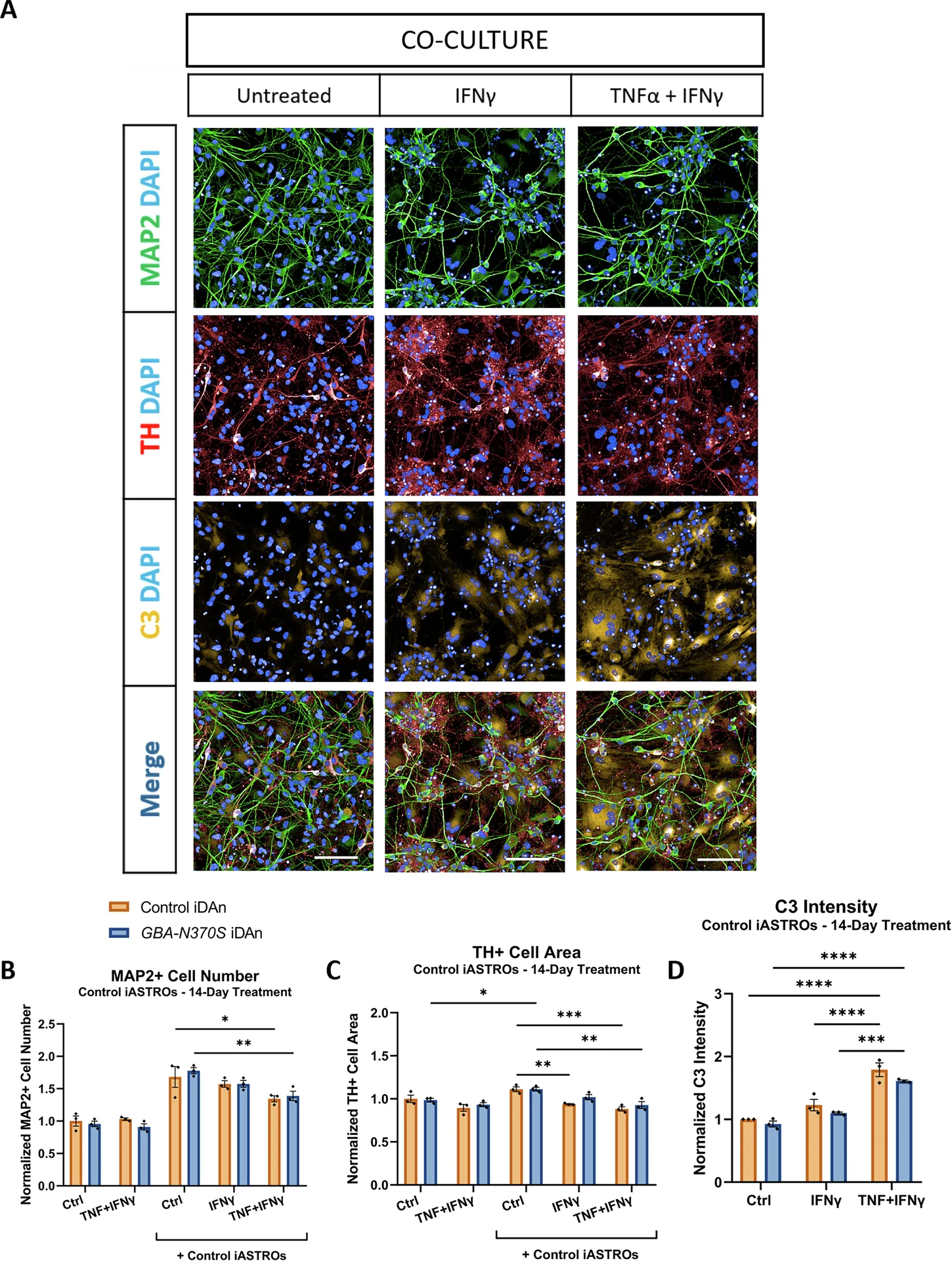
iASTROs treated with TNF and IFN-γ exhibit reduced neuroprotective effects. Control and *GBA-N370S* iDan and iASTROs were co-cultured and directly treated for 14 days with TNF and IFNγ. **A** Representative immunocytochemistry images of control iDAn co-cultured with control iASTROs, untreated or treated with IFNγ or TNF + IFNγ for 14 days, stained for MAP2 (green), TH (red), C3 (yellow) and DAPI nuclear staining (blue). Scale bar: 100 µm. **B–D** Quantification of MAP2+ cell number, TH+ cell area and astrocytic cytoplasmic C3 intensity in control and *GBA-N370S* iDAn, in monoculture or in co-culture with control iASTROs, untreated or treated with 50 ng/mL IFNγ or 30 ng/mL TNF + 50 ng/mL IFNγ for 14 days. Each data point represents 1 iDAn line, averaged in each differentiation to all the conditions either in monoculture or co-cultured with 1–2 lines of control iASTROs, and then averaged across differentiations. For each differentiation, values were normalized to the average of untreated (Ctrl) control iDAn in monoculture. *N* = 3 control and 3 *GBA-N370S* lines for iDAn (3 differentiations), and *N* = 3 control lines for iASTROs (1–2 differentiations per line). Mean ± SEM. The statistical analysis performed was two-way ANOVA with Tukey’s multiple comparison test. *p < 0.05, **p < 0.01, ***p < 0.001, ****p < 0.0001. Not all statistically significant comparisons are shown in the graphs, due to the large number of comparisons with *p* values below 0.05.

iDAn monocultures and co-cultures of control or *GBA-N370S* iDAns with control (Fig. 5) or *GBA-N370S* (Supplementary Fig. 11) iASTROs were treated with IFNγ or TNF + IFNγ for 48 h after establishing the co-culture, and stained for MAP2, TH and C3 14 days later (Supplementary Fig. 2C). The number of MAP2+ cells in the co-cultures decreased after treatment with TNF + IFNγ, indicating reduced neurosupport by iASTROs (Fig. 5A, B and Supplementary Fig. 11A). The area of TH+ cells and the dead cell ratio of the cultures were reduced and increased by TNF + IFNγ, respectively, in support of our MAP2 results (Fig. 5A, C and Supplementary Fig. 11B, D and E). Finally, there was an increase in the intensity of the reactive astrocyte marker C3^40^ in control iASTROs after TNF + IFNγ treatment, indicating a reactive phenotype in the iASTROs (Fig. 5A, D and Supplementary Fig. 11C). Interestingly, while some of these changes were elicited by TNF and IFNγ alone, collectively effects were stronger in the presence of both cytokines (Fig. 5 and Supplementary Fig. 11).

These findings clearly indicate that in co-cultures treated with IFNγ and TNF + IFNγ, iASTROs become reactive and lose their neuroprotective capacities, regardless of genotype.

### TNF and IFN-γ are neurotoxic to iDAns in the presence of primary midbrain astrocytes

**Fig. 6:**
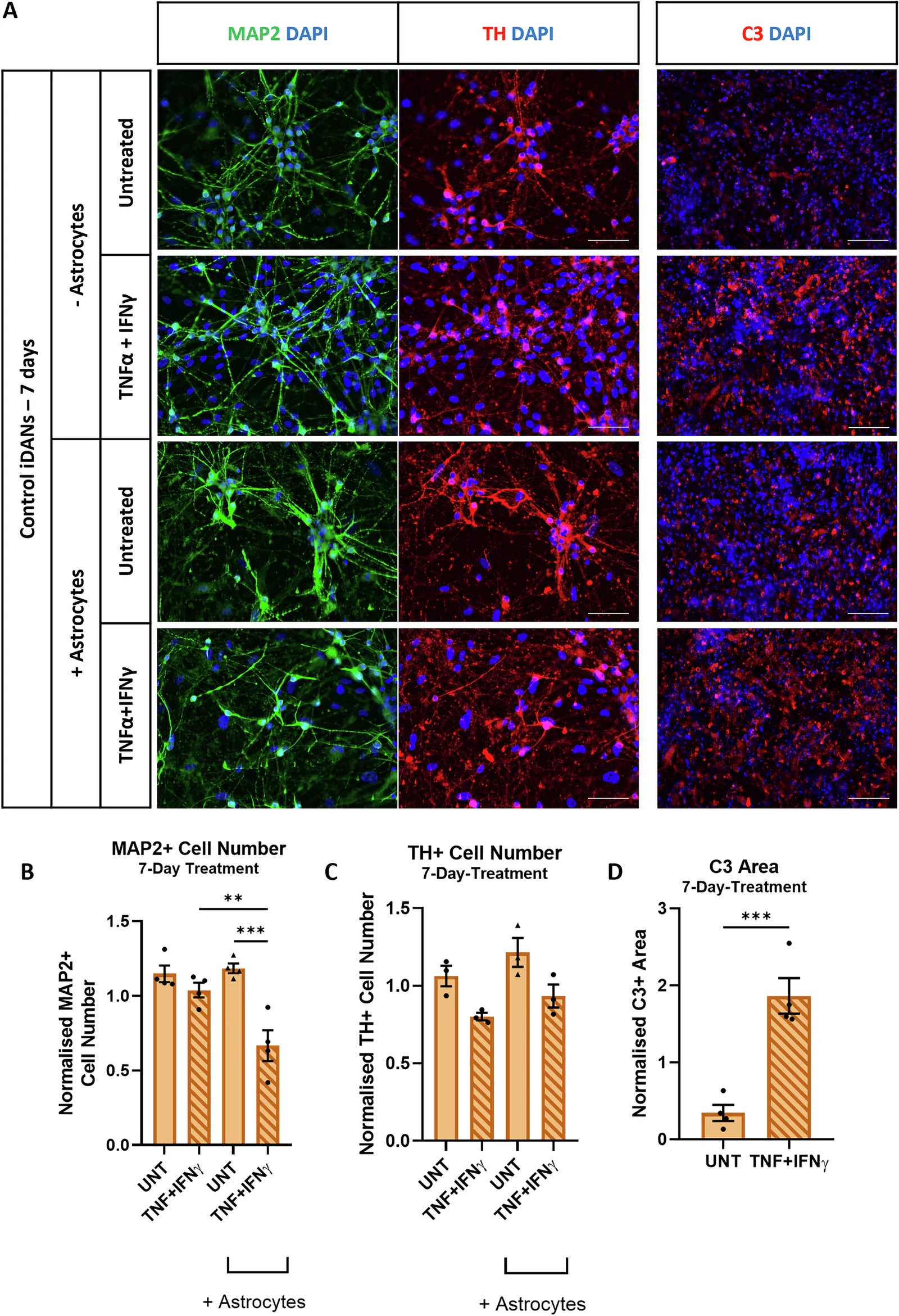
TNF and IFN-γ exert neurotoxic effects on iDAn in the presence of primary midbrain astrocytes. Control iDan and mouse primary midbrain astrocytes were co-cultured and directly treated for 7 days with TNF and IFNγ. **A** Representative immunocytochemistry images of control iDAn co-cultured with control primary astrocytes, untreated or treated with 30 ng/mL TNF + 50 ng/mL IFNγ for 7 days, stained for MAP2 (green), TH (red), C3 (red) and DAPI nuclear staining (blue). Scale bar: 125 µm. **B**–**D** Quantification of MAP2+ cell number, TH+ cell number and astrocytic cytoplasmic C3 intensity in control iDAn, in monoculture or in co-culture with primary midbrain astrocytes, untreated or treated with 30 ng/mL TNF + 50 ng/mL IFNγ for 7 days. Each data point represents 1 iDAn line, averaged in each differentiation to all the conditions either in monoculture or co-cultured with primary midbrain astrocytes, and then averaged across differentiations. For each differentiation, values were normalized to the average of untreated (UNT) control iDAn in monoculture. *N* = 5 control lines for iDAn (2 differentiations). Mean ± SEM. The statistical analysis performed was two-way ANOVA with Tukey’s multiple comparison test. *p < 0.05, **p < 0.01, ***p < 0.001, ****p < 0.0001. Not all statistically significant comparisons are shown in the graphs, due to the large number of comparisons with *p* values below 0.05.

To test whether these results could be observed in an orthogonal model of midbrain astrocytes, namely monocultures and co-cultures of control iDAns with primary midbrain astrocytes, these cultures were treated with a combination of TNF + IFNγ for 7 days (Fig. 6). The number of MAP2+ cells was reduced in co-cultures treated with TNF + IFNγ compared to untreated cells, while in monocultures no significant differences were observed between untreated and treated conditions (Fig. 6A, B). TH+ cell number was also reduced in both mono and co-cultures treated with TNF + IFNγ, compared to untreated groups, but this reduction did not reach statistical significance (Fig. 6A, C). We then analyzed C3 expression specifically in primary midbrain astrocytes (Fig. 6A, D). C3-stained area was significantly increased in the TNF + IFNγ-treated group, compared to the untreated condition. Collectively, these results show that in iDAn and primary midbrain astrocyte co-cultures, combined treatment with TNF and IFNγ induces astrocyte reactivity that is neurotoxic to control iDAns, and that the presence of astrocytes is required to elicit this neurotoxicity.

### Combined exposure to TNF, IFN-γ and PFFs reduces iASTRO neurosupport and induces toxicity

**Fig. 7:**
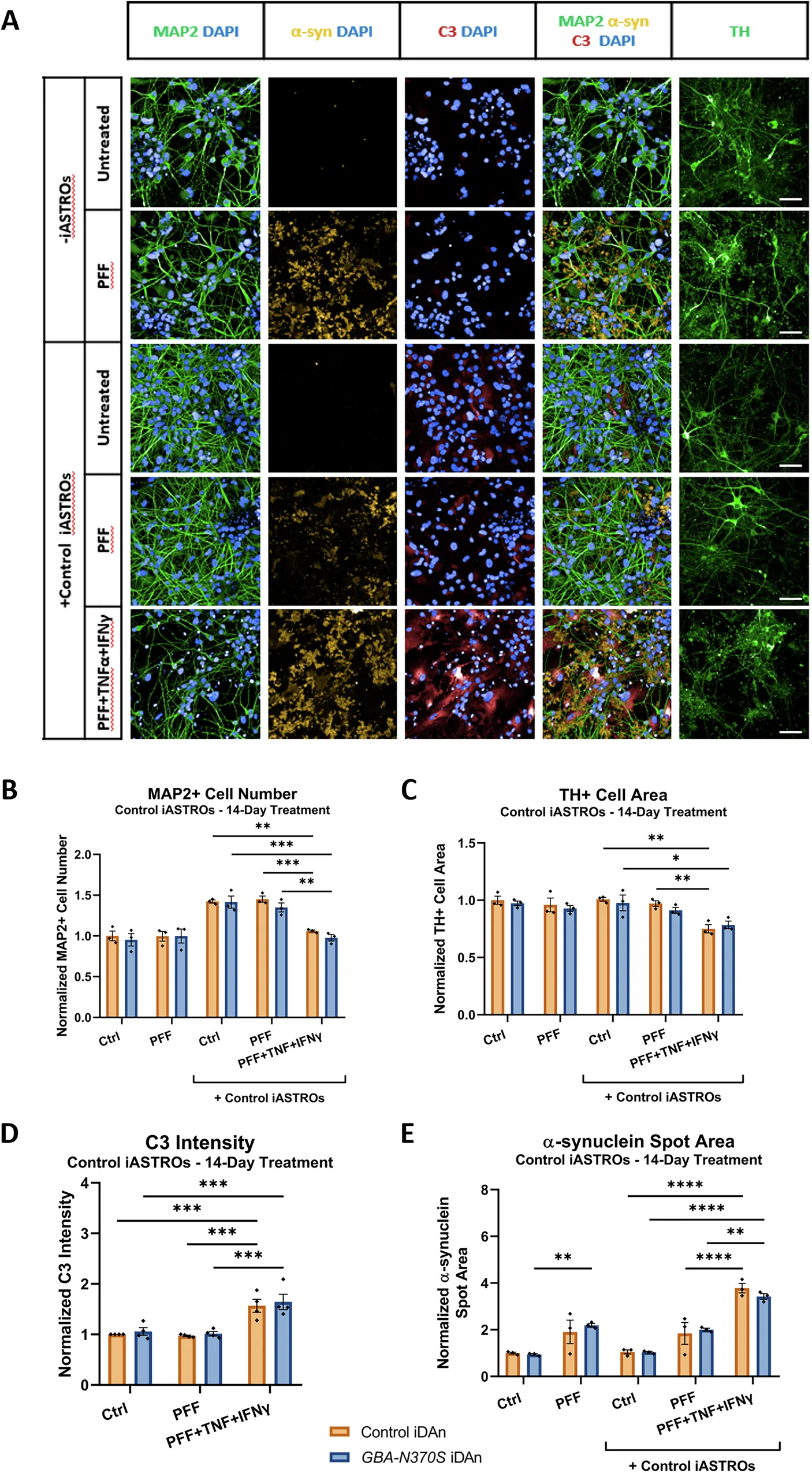
TNF and IFN-γ Treatment promotes PFF-Neurotoxicity by iASTROs. Control and *GBA-N370S* iDan and iASTROs were co-cultured and directly treated for 14 days with α-synuclein PFFs alone or in combination TNF and IFNγ. **A** Representative immunocytochemistry images of control iDAn in monoculture or in co-culture with control iASTROs, untreated or treated for 14 days with α-synuclein PFFs with or without TNF + IFNγ, stained for MAP2 (green), α-synuclein (yellow), C3 (red) and DAPI nuclear staining (blue). Scale bar: 100 µm. B–E Quantification of the number of MAP2+ cells, the cell area of TH+ cells and the area of α-synuclein spots in control and *GBA-N370S* iDAn, and the astrocytic cytoplasmic C3 intensity in iASTROs, in monoculture or in co-culture with control iASTROs, untreated or treated with α-synuclein PFFs (10 μg/mL) or α-synuclein PFFs (10 μg/mL) + TNF (30 ng/mL) + IFNγ (50 ng/mL) for 14 days. Each data point represents 1 iDAn line averaged across differentiations. For each differentiation, values were normalized to the average of untreated (Ctrl) control iDAn in monoculture. *N* = 3 control and 3 *GBA- N370S* lines for iDAn (4–5 differentiations), and *N* = 3 control lines for iASTROs (1–2 differentiations per line). Mean ± SEM. The statistical analysis performed was two-way ANOVA with Tukey’s multiple comparison test. *p < 0.05, **p < 0.01, ***p < 0.001, ****p < 0.0001. Not all statistically significant comparisons are shown in the graphs, due to the large number of comparisons with *p* values below 0.05.

To test the interaction between α-synuclein and TNF + IFNγ, control and *GBA-N370S* iDAns were co-cultured with control (Fig. 7) or *GBA-N370S* iASTROs (Supplementary Fig. 12), treated with α-synuclein PFFs alone or in combination with TNF + IFNγ after 48 h of co-culture and assayed 14 days later (Supplementary Fig. 2C). The number of MAP2+ cells in the co-cultures was significantly reduced only after co-treatment of PFFs and TNF + IFNγ in the presence of iASTROs regardless of the genotype (Fig. 7A, B and Supplementary Fig. 12A). Similarly, the area of TH+ cells was reduced and the dead cell ratio was increased in co-cultures exposed to PFFs and TNF + IFNγ (Fig. 7A, C and Supplementary Fig. 12B, E and F). The C3 intensity of iASTROs was significantly higher when treated with PFFs and TNF + IFNγ compared to untreated co-cultures, but no changes were observed in co-cultures treated with PFFs alone, indicating that even when cocultured with iDAns and being exposed to PFFs for 14 days, iASTROs do not become reactive in response to PFFs (Fig. 7A, D and Supplementary Fig. 12C). We then analyzed α-synuclein spots to investigate whether the capacity to degrade PFFs was greater in co-cultures than in monocultures and whether this was impaired under inflammatory conditions (Fig. 7A, E and Supplementary Fig. 12D). Whilst we observed no changes in the accumulation of α-synuclein between monocultures and co-cultures, we found higher α-synuclein accumulation in co-cultures treated with PFFs and TNF + IFNγ than in cocultures treated with PFFs alone.

In conclusion, these results suggest that α-synuclein PFFs alone do not induce a reactive response in iASTROs in co-cultures. However, combined treatment of α-synuclein PFFs with TNF + IFNγ induces further accumulation of α-synuclein and potentiates the toxic effects of reactive control and *GBA-N370S* iASTROs reducing neuronal support.

### TNF + IFN-γ are required for PFF induced neurodegeneration in primary astrocyte-iDAn co-cultures

**Fig. 8:**
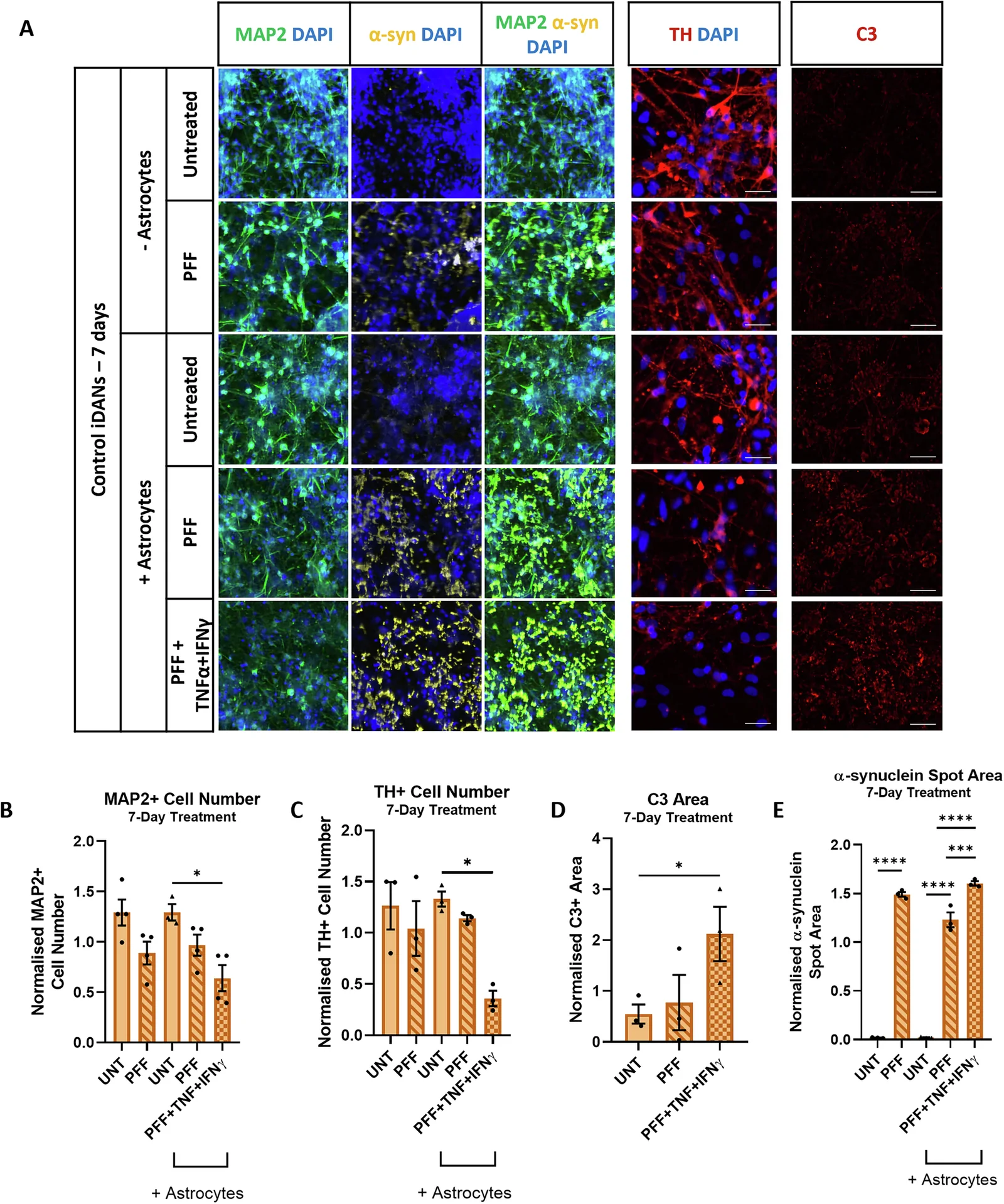
Primary astrocytes cocultured with control iDAN show TNF + IFN-γ dependent PFF induced neurodegeneration. Control iDAn and mouse primary midbrain astrocytes were co-cultured and directly treated for 7 days with α-synuclein PFFs alone or in combination TNF and IFNγ. **A** Representative immunocytochemistry images of control iDAn co-cultured with primary midbrain astrocytes, untreated or treated with α-synuclein PFFs (10 μg/mL) or α-synuclein PFFs (10 μg/mL) + TNF (30 ng/mL) + IFNγ (50 ng/mL) for 7 days, stained for MAP2 (green), α-synuclein (yellow), TH (red), C3 (red) and DAPI nuclear staining (blue). Scale bar: 125 µm. **B**–**E** Quantification of MAP2+ cell number, TH+ cell number and a-synuclein spot area in control iDAn, and astrocytic cytoplasmic C3 intensity, in monoculture or in co-culture with primary midbrain astrocytes, untreated or treated with α-synuclein PFFs (10 µg/mL) or α-synuclein PFFs (10 µg/mL) + 30 ng/mL TNF + 50 ng/mL IFNγ for 7 days. Each data point represents 1 iDAn line, averaged in each differentiation to all the conditions either in monoculture or co-cultured with primary midbrain astrocytes, and then averaged across differentiations. For each differentiation, values were normalized to the average of untreated (UNT) control iDAn in monoculture. *N* = 5 control lines for iDAn (2 differentiations). Mean ± SEM. The statistical analysis performed was two-way ANOVA with Tukey’s multiple comparison test. *p < 0.05, **p < 0.01, ***p < 0.001, ****p < 0.0001. Not all statistically significant comparisons are shown in the graphs, due to the large number of comparisons with *p* values below 0.05.

Control iDAns were cultured alone or with primary midbrain astrocytes and treated with α-synuclein PFFs (in the case of monoculture) or with α-synuclein PFFs alone or in combination with TNF + IFNγ (in the case of co-culture) for 7 days (Fig. 8). The combined treatment of PFFs and TNF + IFNγ resulted in a strong decrease in the number of MAP2+ cells in the presence of primary astrocytes (Fig. 8A, B). The TH+ cell number was also significantly reduced in co-cultures treated with PFFs and TNF + IFNγ compared to untreated co-cultures, while no significant differences were observed in monocultures or in co-cultures treated with PFFs alone (Fig. 8A, C). We next analyzed the C3-stained area in astrocytes to test whether the presence of inflammatory stimuli and PFF treatment induced C3 expression in primary midbrain astrocytes (Fig. 8A, D). C3-positive area was increased in PFF and TNF + IFNγ-treated co-cultures but not in the other conditions, suggesting that the combined treatment induced a reactive response in primary midbrain astrocytes co-cultured with control iDAns. Finally, we analyzed α-synuclein spot area (Fig. 8A, E). As expected, α-synuclein area was significantly increased in both mono and co-cultures treated with PFFs, compared to untreated cultures. Critically, this increase was higher in co-cultures treated with the combination of PFFs and TNF + IFNγ compared to PFF-treated co-cultures (Fig. 8E).

Overall, we found that primary midbrain astrocytes co-cultured with control iDAns and exposed to PFFs and TNF + IFNγ exhibited a reactive phenotype, increased accumulation of α-synuclein and TNF + IFNγ-dependent PFF-induced neurodegeneration.

## Discussion

Here, we present a robust and characterized model of human iPSC-derived midbrain astrocytes (iASTROs) and their neurosupportive and reactive properties in both physiological and inflammatory contexts. We demonstrate that both control and *GBA-N370S* iASTROs exhibit a neurosupportive role toward iDAns, regardless of their genotype, but that this support is compromised under inflammatory conditions, particularly when combined with pathological α-synuclein PFFs stimuli. These findings provide new insights into astrocyte-mediated mechanisms contributing to neuronal vulnerability in PD, especially in the context of *GBA1* mutations.

Our model successfully generated iASTROs derived from both control and *GBA-N370S* iPSCs which exhibit characteristic astrocytic features such as expression of specific astrocytic markers, glutamate uptake, and support to neuronal synaptogenesis, as previously reported by other authors using different protocols^41–43^. However, many of the iPSC-derived astrocyte protocols available fail to generate region-specific cells such as ventral midbrain astrocytes^27,28^. By using the protocol developed by Crompton et al.^34^, we were able to generate iPSC-derived ventral midbrain astrocytes that clustered closer to substantia nigra human astrocytes rather than human cortical astrocytes as revealed by transcriptomic profiling, further supporting their midbrain identity and their role in PD given the selective vulnerability of the SNc in PD^44,45^.

Neuroinflammation is well known to be a key player in PD, and that crosstalk with microglia shifts astrocytes towards a reactive and potentially neurotoxic phenotype where they lose their homeostatic astrocytic functions^46^. Furthermore, it has also been reported that astrocytes can directly respond to inflammatory stimuli such as the TNF, IL-1α and C1q cocktail, which has been widely studied in neuroinflammation^47,48^. However, in our study we exposed control iASTROs to different treatments for 24 h. We found that exposure to TNF and IFNγ led iASTROs to the biggest transcriptional change, which triggered significant activation of pathways related to immune response and calcium transport and homeostasis, while treatment with TNF, IL-1α and C1q showed more modest transcriptional changes.

Among the pathways dysregulated by proinflammatory stimulation with TNF and IFNγ, calcium transport and homeostasis emerged as a key underexplored feature of astrocytic reactivity. Functionally, *GBA-N370S* iASTROs exhibited reduced ATP-induced calcium release compared to control iASTROs, in line with previous findings showing calcium dysregulations in iPSC-derived astrocytes from *GBA-N370S* donors^49^. Calcium response has also been reported to be affected in different models of PD-related mutations where both neurons and astrocytes display dysregulated calcium release^17,32,50,51^. As previously reported by Panattoni et al.^52^ who described an increase in calcium release from spinal cord astrocytes after exposure to different cytokines and/or LPS in a model of organotypic slices, we also observed an increase in calcium release in response to inflammatory cytokines both in *GBA-N370S* iASTROs (which reached levels comparable to those of the controls) and primary midbrain astrocytes. Importantly, blocking the calcium response did not appear to affect the inflammation profile of the astrocytes, pointing towards a role for inflammation in calcium release. Untangling the relationship between calcium release, inflammation, and neuronal support represents an enormous challenge. Because our system combines lysosomal enzyme inhibition (both genetic and chemical), inflammatory cytokine exposure, and α-synuclein pathology, experimentally isolating calcium-specific effects in the presence of GBA mutations or CBE, together with cytokine stimulation represents a substantial technical and interpretational challenge, particularly in co-culture where cell-autonomous and non–cell-autonomous mechanisms interact. Using an inverse approach, we inhibited calcium signaling pharmacologically with APB but did not observe detectable changes in the relevant inflammatory readouts. These findings suggest that inhibition of this pathway alone is insufficient to alter the phenotype, indicating that any contribution of calcium dysregulation to neurotoxicity is likely context-dependent and mechanistically complex. It was very interesting to observe the phenocopy of iDAns in the ER-mitochondria separation in *GBA-N370S* iASTROs, pointing towards a common dysregulation of organellar contact sites in cells harboring the mutation, which have been shown to play crucial roles in cellular homeostasis^53^. More interestingly, we report for the first time to our knowledge an increase in the proximity between mitochondria and the lysosome in iASTROs, all of which could be responsible for the observed phenotypes and is in keeping with our recent findings in *GBA-N370S* iDAns^54^.

To assess the functional consequences of astrocyte reactivity, we investigated how TNF + IFNγ inflammatory stimuli affect the neuroprotective properties of iASTROs. Prolonged exposure to TNF + IFNγ reduced the number of MAP2+ and TH+ cells in iDAn-iASTRO co-cultures, which was accompanied by increased expression of C3 in iASTROs, a reactive astrocyte hallmark known to mediate astrocytic reactivity^46,55^. In line with these findings, a study described an iPSC-derived neuron and astrocyte co-culture model where astrocytes underwent a reactive phenotype after TNF and IL-1β exposure, affecting to neuron axonal growth^56^. Our results suggest that exposure to proinflammatory cytokines shifts both control and *GBA-N370S* iASTROs towards a non-supportive toxic state, in line with the emerging concept of reactive astrocytes in PD pathology^40^.

We further explored the influence of α-synuclein PFFs alone or in combination with proinflammatory cytokines on astrocyte reactivity and neurotoxicity. PFFs alone failed to evoke a reactive response in iASTROs or to impair neuron survival in either mono or co-culture, even after prolonged exposure, likely suggesting that ventral midbrain astrocytes are sufficient to buffer PFF insults and protect neurons from them, as described by Yang et al.^57^. Interestingly, PFF treatment induced α-synuclein accumulation in *GBA-N370S* neuronal cultures. However, when neurons were co-cultured with astrocytes this was reduced to non-significant levels, highlighting that astrocytes can indeed support the degradation of α-synuclein in homeostatic conditions, and that *GBA-N370S* neurons are probably more intrinsically vulnerable to insult. In contrast, Chou et al.^58^ found that α-synuclein PFFs alone were able to induce a reactive phenotype in primary human midbrain astrocytes, which, when co-cultured with SH-SY5Y cells, resulted neurotoxic. However, the PFF + TNF + IFNγ treatment induced a significant reduction in MAP2+ and TH+ cells in co-cultures. Interestingly, α-synuclein burden and astrocytic C3 expression was most pronounced upon this combined treatment, further evidencing a synergistic link between α-synuclein aggregation and astrocytic inflammation in PD and supporting a “death by a thousand cuts” hypothesis in the disease^59^. Importantly, we observed similar inflammatory-induced neurotoxicity in an independent control iDAn-primary midbrain astrocyte co-culture, with increased astrocytic C3 expression and α-synuclein accumulation after 7-day PFF + TNF + IFNγ treatment. In our experimental co-cultures, primary mouse cortical astrocytes exhibited a significantly greater neurotoxic effect on neurons than iASTROs. One possible explanation is that astrocytes differentiated from iPSCs often retain an immature developmental phenotype, as iPSC-derived astroglia typically reflect early developmental stages absent of the full in vivo maturation signals found in the brain parenchyma, which can influence key functional properties relevant to neurotoxicity and support of neurons^60^. Additionally, our primary cortical astrocytes were cultured in medium containing serum, a condition that has been widely shown to induce a “reactive astrocyte-like” phenotype in vitro; this increased basal astrocyte reactivity may underlie the enhanced neurotoxicity observed relative to the iPSC-derived cells^61^. Additionally, iASTROs were seeded at 11,000 cells per well, whereas primary astrocytes were seeded at 400 cells per well; this substantial difference in astrocyte density may contribute to the reduced neuronal support and stimulation observed under control co-culture conditions with primary astrocytes. These parallel findings in primary cells support the validity of our iPSC-derived midbrain astrocyte model, demonstrating that cytokine-induced astrocytic reactivity is sufficient to drive α-synuclein-mediated neurodegeneration. Whilst it is interesting to note that, in this context, we found no specific effects for the *GBA-N370S* mutation, one might speculate that extended co-cultures with *GBA-N370S* iASTROs may be able to reveal reduced neurosupport and/or increased neurotoxicity. It is also interesting to hypothesize that perhaps *GBA-N370S* iASTROs could be more sensitive to other signaling cues from microglia currently missing in our co-cultures, which could be explored in future studies.

Collectively, our data provide evidence of a model in which fully neurosupportive astrocytes undergo a shift toward a non-supportive potential neurotoxic phenotype in the presence of proinflammatory cues. Among the features of this reactive phenotype, we find intracellular calcium dysregulation and impaired neuronal support. The *GBA-N370S* mutation exacerbates these effects by increasing astrocyte vulnerability to inflammatory stimuli and promoting enhanced ATP-induced ER calcium release, a finding consistent with previous studies implicating the *GBA-N370S* mutation in lysosomal and calcium homeostasis dysfunction^19,62^. Our data links these phenotypes to aberrant organellar separation and perhaps contact site modulation. However, genotype seems not to play a major role in the loss of astrocytic neurosupport observed in co-culture, suggesting a genotype-independent effect, or increased latency for some phenotypes. These findings provide mechanistic insight into how cell-autonomous and non-cell-autonomous mechanisms, particularly those involving astrocytic reactivity, contribute to DAn vulnerability in PD.

Altogether, these results provide new evidence of the role of gliosis in neurodegenerative diseases and could help to design new anti-inflammatory drugs targeting astrocytes and thus preventing neurotoxicity.

## Methods

### iPSC-derived dopaminergic cultures

iPSC lines used in this study were derived from human skin biopsy fibroblast acquired with informed consent and ethical approval (Ethics committee: National Health Service, Health Research Authority, NRES Committee South Central, Berkshire, UK, REC 10/H0505/71) and are commercially available at EBiSC. All ethical guidelines were followed. iPSCs were differentiated into DAns following a previously described protocol^63^. iPSCs from healthy (SFC065-03, SFC67-03, SFC156-03, SFC840-03 and SFC856-03) and *GBA-N370S* (MK071, MK082, MK088, SFC848-03 and SFC871-03) donors (Table 1) were plated at a density of 150,000 cells/cm^2^ on Geltrex (Life Technologies, A1413302) and cultured until confluent in an incubator at 37 °C and 5% CO_2_^64^. Unless otherwise stated all the cells in this study were grown at 37 °C and 5% CO_2_. Cells were then grown for 11 days in KO-KSR media containing Knockout DMEM™ (KO-DMEM (Life Technologies, 10829018)) supplemented with 15% Knockout™ Serum Replacement (KSR (10828010, Life Technologies)), 1% L-Glutamine (Thermo Fisher Scientific, 25030024), 0.02% 2-Mercaptoethanol (Life Technologies Ltd, 21985023) and 1% Penicillin-Streptomycin (Life Technologies Ltd, 15140122). KO-KSR media was gradually shifted to NNB medium containing Neurobasal™ Medium (Life Technologies Ltd, 21103049) supplemented with 0.5% N2 Supplement (Life Technologies Ltd, 17502048), 1% B-27™ Supplement without Vitamin A (Life Technologies Ltd, 12587010), 1% L-Glutamine and 1% Penicillin-Streptomycin, starting on day 5 of the differentiation. Different combinations of LDN-193189 (100 nM (Sigma Aldrich, SML0559)), SB-431542 (10 μM (APExBIO, A8249-APE)), SHH-C24II (100 ng/mL (R&D Systems, 1845-SH)), Purmorphamine (2 µM (Tocris, 4551/10)), FGF8a (100 ng/mL (Cambridge Bioscience Ltd, Z03172)) and CHIR-99021 (3 µM (Tocris, 4423/10)) were used to induce ventral midbrain patterning of the iPSCs as previously described^64^. On day 11, media was changed to NB media (containing Neurobasal™ medium supplemented with 1% B-27™ Supplement without vitamin A, 1% L-glutamine and 1% Penicillin-Streptomycin) supplemented with CHIR-99021 (3 µM, until day 13), BDNF (brain-derived neurotrophic factor; 20 ng/mL (Peprotech, 450-02)), Ascorbic Acid (0.2 mM (Sigma-Aldrich, A4544)), GDNF (glial cell line-derived neurotrophic factor; 20 ng/mL (Peprotech, 450-010), TGFβ3 (transforming growth factor type β3, 1 ng/mL (Peprotech, 100-36E)), dibutyryl-cAMP (0.2 mM (Sigma-Aldrich, D0267)), and DAPT (10 mM, Abcam, ab120633) and cells received this medium daily for 9 days. On day 20, cells were dissociated using Accutase (Life Technologies) and then replated at the cell density of 40,000 cells/well on Geltrex in a half-area 96-well plate (Greiner) in final differentiation medium (NB supplemented with BDNF, GDNF, TGFb3, DAPT, db-cAMP and Ascorbic Acid in the above-mentioned concentrations supplemented with 10 µM ROCK inhibitor (Y-27632 (APExBIO, A3008-APE)). On day 22, cell culture was treated for an hour with 1 μg/mL Mitomycin C (Abcam, ab120797) in Neurobasal™ Medium to remove non-differentiated cells. Cells were then washed and cultured in the final differentiation medium, with half-medium changes every Monday, Wednesday, and Friday until experiments were performed at DIV35.

**Table 1:** iPSC lines used in this study

Genotype	iPSC line name	Reprogramming method	Age	Sex	Publication	EBiSC ID
Healthy control	SFC065-03-03	Cytotune (Sendai)	65–69	Male	^17^	STBCi057-A
Healthy control	SFC067-03-01	Cytotune (Sendai)	70–74	Male	^70^	STBCi105-A
Healthy control	SFC156-03-01	Cytotune (Sendai)	75–79	Male	^70^	STBCi101-A
Healthy control	SFC840-03-03	Cytotune (Sendai)	65–69	Female	^15^	STBCi026-A
Healthy control	SFC856-03-04	Cytotune (Sendai)	75–79	Female	^71^	STBCi063-A
GBA-N370S	MK071-3	Retrovirus	80–84	Female	^15^	UOXFi001-B
GBA-N370S	MK082-26	Retrovirus	50–54	Male	^70^	UOXFi002-A
GBA-N370S	MK088-1	Retrovirus	45–49	Male	^15^	UOXFi003-A
GBA-N370S	SFC848-03-02	Cytotune (Sendai)	65–69	Male	^11^	STBCi042-A
GBA-N370S	SFC871-03-09	Cytotune (Sendai)	70–74	Female	^11^	STBCi084-C

### iPSC-derived midbrain astrocyte cultures

iPSCs were patterned towards a midbrain fate as described^34^ in Supplementary Fig. 2. When cells reached confluency in 6-well plates, media was replaced with Day 0–10 media containing NNB (Neurobasal™ Medium supplemented with 0.5% N2 Supplement, 1% B-27™ Supplement without Vitamin A, 1% L-Glutamine and 1% Penicillin-Streptomycin) supplemented with LDN-193189 (100 nM), SB-431542 (10 µM), SHH-C24II (200 ng/mL) and CHIR-99021 (800 nM). Between days 3-4 and 20, when ventral midbrain neural progenitor cells (vmNPCs) reached confluency, cells were single-cell passaged in a 1:2 ratio. On Day 11, the media was fully replaced with a media containing NNB supplemented with GDNF (20 ng/mL), BDNF (20 ng/mL) and Ascorbic Acid (200 µM). After Day 20, vmNPCs were passaged at a higher ratio (1:3 or 1:4). On Day 30 differentiation and expansion into vmAPCs was started by fully replacing media with APC media containing ASTRO media (Modified Serio medium, ie Advanced DMEM/F-12 (Life Technologies Ltd, 12634028) supplemented with 1% MEM non-essential amino acids (NEAA (Life Technologies Ltd, 11140050)), 1% GlutaMAX™ Supplement (Life technologies Ltd, 35050061), 1% N2 Supplement, 0.4% B-27™ Supplement without Vitamin A and 1% Penicillin-Streptomycin) supplemented with EGF (20 ng/mL (Peprotech, AF-100-15)) and hLIF (20 ng/mL (Peprotech, 300-05)). When cells reached confluency, they were single cell passaged in a 1:2–1:8 ratio and plated in APC media supplemented with 10 µM ROCK inhibitor. vmAPCs were fed every 2–4 days and passaged as required until day 90 of the protocol. After day 90, to generate mature iASTROs, media was fully replaced with MAT media containing ASTRO media supplemented with BMP4 (20 ng/mL (Peprotech, AF-120-05ET)) and hLIF (20 ng/mL (Peprotech, 300-05)) feeding them every 3 days and passaging them upon confluency. After 7 days in MAT media, iASTROs were considered fully mature, and at Day 100 iASTROs were plated into the final assay layout for mono- and co-culture assays.

### Primary midbrain astrocyte cultures

Primary cells used in this study were derived from C57Bl6 mouse pups following ethical protocols of the Ethics committee for animal studies of the University of the Basque Country (Reference M20/2022/254) approved by the local authority namely the Basque Government and in compliance with the EU directive 2010/63/EU. All ethical guidelines were followed. Primary cultures of midbrain astrocytes were prepared from newborn (P0-P2) C57BL6 mice as described before^65^. The brain hemispheres were extracted and separated, the meninges were removed, and the midbrain was dissected.

Midbrain tissue was digested with 0.25% trypsin (Gibco, A325200056) and 0.4% deoxyribonuclease (Merck, D5025-15KU) for 5 min at 37 °C. After digestion, the enzymatic reaction was stopped by adding Neurobasal™ medium supplemented with 10% Fetal Bovine Serum (Biowest, S140B-100) and centrifuged at 400 × *g* for 5 min. The medium was aspirated, 2 mL of dissection media (Neurobasal™ Medium supplemented with 10% FBS, 1% L-Glutamine and 1% Penicillin-Streptomycin) was added, and the pellet was gently resuspended. The cellular suspension was dissociated mechanically by passing it through 23- and 25-gauge needles. Finally, it was filtered through a 40 μm nylon cell strainer (Falcon^®^, 352340) to filter out large cell clumps and tissue debris. The solution was centrifuged at 400 × *g* for 5 min, and the resulting cells were seeded in astrocyte medium (Neurobasal™ Medium supplemented with 15% FBS, 1% L-Glutamine and 1% Penicillin-Streptomycin) onto 6-well plates.

When confluency was reached, cells were trypsinized, and astrocytes were plated onto half-area 96-well plates in different densities depending on the experiment.

### iPSC-derived dopaminergic neuron and primary mouse astrocyte co-culture

DIV35 iDAns were co-cultured with primary midbrain mouse astrocytes. The differentiation medium was removed, and neurons were fed with 25 µL per well of a 50/50 mixture (co-culture medium) of differentiation medium and astrocyte medium. Astrocytes were trypsinized, centrifuged and plated in 25 µL of the co-culture medium with 10 µM ROCK inhibitor (Biogen, Y-27632) on top of the neurons at a density of 300 astrocytes per well. Co-culture was treated 48 hours after as needed and half-medium changed every 2 days for 1 week.

### Human iPSC-derived midbrain astrocyte and dopaminergic neuron co-culture

Mature DIV 97-100 iASTROs were plated on top of DIV 35 iDAns cultured in half-area 96-well plates. On the day of co-culture, all media was removed from iDAns and 25 μL iDAn maturation media were added to each well. Then, iASTROs were lifted, spin down, resuspended in MAT media supplemented with 10 μM ROCK inhibitor (Y-27632) and counted. iASTROs were diluted to obtain a cell suspension of 3.2 × 10^5^ cells/mL, and 25 μL of this iASTRO suspension were added to the desired wells containing iDAns, so that the final iASTRO number was 8 × 10^3^ cells/well. In the wells where no iASTROs were added, 25 μL of MAT media supplemented with 10 μM ROCK inhibitor (Y-27632) were added to iDAns. 48 h after replating, a full media change was done, or cells were treated, using 50% medium (50% iDAn maturation medium + 50% astrocyte MAT medium) in both cases.

### Preparation of α-synuclein pre-formed fibrils

Monomeric human and mouse α-synuclein were acquired from rpeptide. Endotoxin removal columns (ThermoFisher/Pierce) were used to remove endotoxin from monomeric human α-synuclein, and the Pierce™ Chromogenic Endotoxin Quant Kit was used to ensure endotoxin removal below 0.01 EU/ml. 5 mg/mL α-synuclein monomers were shaken at 37 °C and 250 rpm for 7 days to induce aggregation and were sonicated to obtain pre-formed fibrils^66^. PFFs were observed in an electron microscope to verify correct size (an average of 100 nm length) and morphology (Supplementary Fig. 1).

### Cell treatments

All cell treatments were done with the following reagents and concentrations: PFFs 10 μg/mL (homemade), TNF 30 ng/mL (Peprotech), IFNγ 50 ng/mL (Biolegend), C1q 400 ng/mL (Sigma-Aldrich), and Il1α 3 ng/mL (Peprotech).

### qPCR

Cells were harvested and RNA was extracted using an RNA extraction kit (RNeasy Mini Kit, Qiagen). cDNA was synthesized using the High-Capacity RNA-to-cDNA™ Kit (Applied Biosystems). Primers used for RT-qPCR analysis are shown in Table 2. qPCR was performed using the FAST SYBR Green master mix (Applied Biosystems) on StepOnePlus™ System (Applied Biosystems). Data were normalized to the housekeeping gene *GAPDH* which generally did not vary more than 2 cycles in each experiment.

**Table 2:** Primers used for qPCR

Gene	Forward primer (5′-3′)	Reverse primer (3′-5′)
C3	TACAACGTGGAGGCCACATC	ACGGGAGGCACAAAGTCAAA
CCL5 (RANTES)	CCTGCTGCTTTGCCTACATTGC	ACACACTTGGCGGTTCTTTCGG
CD44	CTGCCGCTTTGCAGGTGTA	CATTGTGGGCAAGGTGCTATT
GAPDH	TCATCATCTCTGCCCCCTCT	TCATGGATGACCTTGGCCAG
GFAP	AGAAGCTCCAGGATGAAACC	AGCGACTCAATCTTCCTCTC
ITPR1	GTGACAGGAAACATGCAGACTCG	CAGCAGTTGCACAAAGACAGGC
ITPR3	GGGGTAATCATCGACACCTTC	GTTATCAAACTTGTCCCTCTCCA
KI67	GAAAGAGTGGCAACCTGCCTTC	GCACCAAGTTTTACTACATCTGCC
NFKB1	GCAGCACTACTTCTTGACCACC	TCTGCTCCTGAGCATTGACGTC
S100B	GGGAGACAAGCACAAGCTGAA	CATTCGCCGTCTCCATCATTG
SOX2	TACAGCATGTCCTACTCGCAG	GAGGAAGAGGTAACCACAGGG
TH	CGAGCTGTGAAGGTGTTTGA	CACGAAGTACTCCAGGTGG

### Immunocytochemistry

Cells were fixed in 4% paraformaldehyde (PFA) for 5 min. Cells were then permeabilised in PBS with 0.1% Triton X-100 and blocked in 10% normal donkey serum (NDS) (VWR, S2170-500) for 1 h at room temperature (RT). Primary antibodies (Table 3) were incubated overnight at 4 °C in tris-buffered saline with 0.05% Tween®20 (TBS-T). AlexaFluor-conjugated secondary antibodies were incubated for 1 h in dark at RT in TBS-T. For nuclear staining, cells were counterstained with DAPI (Avantor) in TBS-T for 5 min at RT. Images were captured with the Opera Phenix (Perkin Elmer, confocal) or the EVOS M7000 (ThermoFisher, epifluorescence) as appropriate. As a negative control, a sample was treated with all reagents except for primary antibody to verify that no non-specific binding was observed.

**Table 3:** Primary antibodies used for immunocytochemistry

Marker	Host species	Catalog #	Dilution	Company
GFAP	Chicken	Ab4674	1:500	Abcam
GFAP	Chicken	AB_2313547	1:1000	AvesLabs
S100B	Mouse	SAB4200671	1:250	Sigma-Aldrich
TH	Rabbit	AB152	1:500	Millipore
TH	Sheep	AB1542	1:500	Millipore
MAP2	Chicken	ab92434	1:1000	Abcam
Homer1	Rabbit	160003	1:500	Synaptic Systems
α-synuclein (4D6)	Mouse	ab2434	1:1000	Abcam
NF-κB p65 (D14E12)	Rabbit	8242	1:400	Cell Signaling
Foxa2	Goat	AF2400	1:250	R&D Systems
C3d	Rabbit	A006302-2	1:500	Agilent

### ATP-induced calcium release

iASTROs plated on half-area 96-well plates were incubated with 2.5 mM Fura-2 QBT Calcium Explorer Kit (Molecular Devices, R8197) solution in MAT media for 1.5 h at 37 °C. After incubation, plates were read using a FlexStation 3 Multi-Mode Microplate Reader (Molecular Devices) programmed for a total recording time of 180 s, injecting a final working solution of 100 µM ATP at 30 s.

For primary midbrain astrocytes, 1000 cells/well were plated on half-area 96-well plates and incubated with Fluo-4 AM (ThermoFisher, F141201) calcium dye diluted 1:1000 in HBSS (without CaCl_2_ and MgCl_2_) (Gibco, 11530476) supplemented with 20 mM HEPES buffer (pH 7.4) (Gibco, 15630056) for 30 min at 37 °C. Fluo-4 solution was then removed, and cells were washed once with HBSS (without CaCl_2_ and MgCl_2_) supplemented with 20 mM HEPES buffer (pH 7.4) and 25 µL of HBSS (without CaCl_2_ and MgCl_2_) supplemented with 20 mM HEPES buffer (pH 7.4) was added to each well. Plates were recorded at 30 Hz with an EVOS M7000 (ThermoFisher) for 60 s, injecting a final working solution of 100 µM ATP at 5 s.

For the analysis, 340/380 nm absorbance ratios (for Fura-2) and mean intensity of selected cells (for Fluo-4) were measured, and the peak amplitude of the trace was calculated.

### Store-operated calcium entry (SOCE)

iASTROs plated in half-area 96-well plates were incubated in 2.5 mM Fura-2 AM dilution supplemented with 100 mM Thapsigargin (Enzo, BML-PE180) for 1.5 h at 37 °C. After, cells were washed 3 times with HBSS (without CaCl_2_ and MgCl_2_) supplemented with 20 mM HEPES buffer (pH 7.4) and 50 µL of HBSS (without CaCl_2_ and MgCl_2_) supplemented with 20 mM HEPES buffer (pH 7.4) was added to each well. Plates were read using a FlexStation 3 Multi-Mode Microplate Reader (Molecular Devices) programmed for a total recording time of 180 s, injecting a final working solution of 2.5 mM CaCl_2_ at 30 s.

For the analysis, 340/380 nm absorbance ratios were measured, and the peak amplitude of the trace was calculated.

### Bulk RNA sequencing and data processing

After 24 h of treatment, total RNA from 3 different DIV100 control iASTRO lines was was purified with the QIAGEN RNeasy Mini Kit and RNA integrity was determined by Novogene using the Agilent 5400 Fragment Analyzer. Non-stranded cDNA libraries were produced with polyA enrichment and sequenced on the Illumina NovaSeq 6000 instrument (PE 150). The sequencing data was processed and analyzed with Salmon (1.10.0), TrimGalore (0.6.10), R (4.4.2) and python (3.12.11).

### Production of raw count matrices

For bulk RNA-seq data (stem cell-derived astrocytes and DaNs), the raw sequencing reads produced by Hedegaard et al.^67^, di Domenico et al.^27^, Krauskopf et al.^68^, and Semeano et al.^69^ were obtained from the Gene Expression Omnibus (accession codes GSE149598, GSE116124, GSE196190, GSE190686, respectively). Adaptor trimming was performed with TrimGalore and sequencing reads were aligned onto a human reference transcriptome (Ensembl GRCh38 release 97 - including haplotypes and scaffolds) with Salmon. Transcript-level count data was then summarized to gene-level with the R package tximport (1.32.0).

For scRNA-seq data (post-mortem human brain tissues), we downloaded sample-specific UMI-barcode matrices prefiltered for lowly expressed genes and poor-quality nuclei from the Gene Expression Omnibus (accession code GSE140231). Cell type annotations were kindly shared by the authors of the original publication (Agarwal et al.^38^) in personal correspondence. A unified count matrix was constructed using the Python library Scanpy (1.11.3) and pseudobulk profiles were then generated for each combination of donor ID and cell type using in-house R functions.

### Analysis of sample similarity

For all analyses of sample similarity, we considered only the bulk RNA-seq samples corresponding to untreated biological conditions and the protein-coding genes detected with at least 10 raw counts in at least 3 samples. For comparison with post-mortem human brain tissues, raw counts for iASTROs and selected human brain pseudobulk profiles (either substantia nigra and cortical astrocytes or substantia nigra cell types) were combined in a single matrix, keeping only the genes detected in both datasets. We systematically applied regularized log transformation to produce homoscedastic count data on the log2 scale and normalized for library size using the R package DESeq2 (1.44.0). The batch effect associated with dataset ID was removed from the transformed data using the R package limma (3.60.6). The batch-corrected, rlog-transformed count matrix was used to produce a heatmap of the sample-to-sample Euclidean distances or a principal component plot based on the 1,000 most variable genes using the R packages pheatmap (1.0.12) or DESeq2. Formal statistical testing on the Euclidean distance matrices was performed using the R packages stats (4.4.2) and vegan (2.7.1).

### Proximity ligation assay (PLA)

ER-lysosome (ER-LYS), ER-mitochondria (ER-MIT), and mitochondria-lysosome (MIT-LYS) interactions were measured using the Duolink kits (Sigma) as previously reported^17^. The used antibodies and their concentrations are listed in Table 4. After 4% PFA fixation, cells were incubated with Duolink block solution at 37 °C for 1 h and then with conjugates diluted in Duolink PLA diluent overnight at 4 °C. Samples were washed with tris buffered saline (TBS) containing 0.05% Tween-20 (TBS-T) and incubated with Duolink ligation reagents for 1 h at 37 °C, washed four times with TBS-T, and then incubated with Duolink amplification reagents for 2.5 h at 37 °C. Cells were washed with Duolink washing buffer and incubated with AlexaFluor-conjugated secondary antibodies and DAPI diluted in TBS for 1 h at RT. Finally, cells were washed with Duolink washing buffer and images were captured with an EVOS M7000. As a negative control, a sample was treated with all reagents except for the ligase in the ligation step to verify no spurious interactions were observed.

**Table 4:** Primary antibodies used for PLA

Marker	Host specie	Catalog #	Dilution	Company
IP3R3	Rabbit	AB9076	1:100	Millipore
TOMM20	Rabbit	ab220822	1:100	Abcam
VDAC1	Mouse	ab14734	1:100	Abcam
Lamp1	Mouse	sc-20011	1:100	Santa Cruz

### Statistical analysis

All data are represented as mean ± SEM (standard error of the mean), unless stated otherwise. The statistical tests used in each figure are listed in the legends. All graphs were produced using the GraphPad Prism v10.0.3 software, which was also used to perform the statistical analyses. Unless stated otherwise, each data point represents one cell line, averaged across technical replicates and multiple differentiations, details of which can be found in each figure legend. When normalized values are shown, these are normalized to the average of the control lines in the control/untreated condition, as specified in the figure legends. The number of lines used per genotype, as well as the number of differentiations used, are specified in the figure legends.

The details of the specific statistical tests and post-hoc tests for multiple comparisons used in each experiment can be found in the figure legends. *p*-values > 0.05 were considered not significant (ns). *p ≤ 0.05, **p ≤ 0.01, ***p ≤ 0.001, ****p ≤ 0.0001 for all tests.

### Ethics approval and consent to participate

All studies comply with the Code of Ethics of the World Medical Association (Declaration of Helsinki). iPSCs were derived from human skin biopsy fibroblasts from patients that had provided informed consent (Ethics committee: National Health Service, Health Research Authority, NRES Committee South Central, Berkshire, UK, REC 10/H0505/71).

## Supplementary information

Supplementary information

## Data Availability

The raw sequencing data (fastq files) for the iASTRO samples is available from the Sequence Read Archive under the BioProject accession number PRJNA1303578. The corresponding processed data (Salmon’s quant.sf files) is available from the Gene Expression Omnibus. The scripts used to perform all transcriptomic analyses are publicly available on GitHub. All other data generated or analyzed during this study are included in this published article (and its supplementary information files), or are available from the corresponding author on reasonable request.

## Code availability

The code used for the analysis of the RNAseq data in this paper is available at https://github.com/naroa-ibarra/2022-Astrocyte-RNAseq/tree/main and https://github.com/naroa-ibarra/2022-Astrocyte-RNAseq/blob/main/scripts/run_dgea_gsea.R
