# Supplementary material for: Reactive astrocytes mediate toxicity in iPSC derived dopaminergic neurons
Source: NPJ Parkinsons Dis. 2026 Jun 12;12:240. doi: 10.1038/s41531-026-01378-9 (PMC13635230; doi:10.1038/s41531-026-01378-9)
Supplement: Supplementary file 1 [file 41531_2026_1378_MOESM1_ESM.pdf]

Supplementary Figure 1:

A

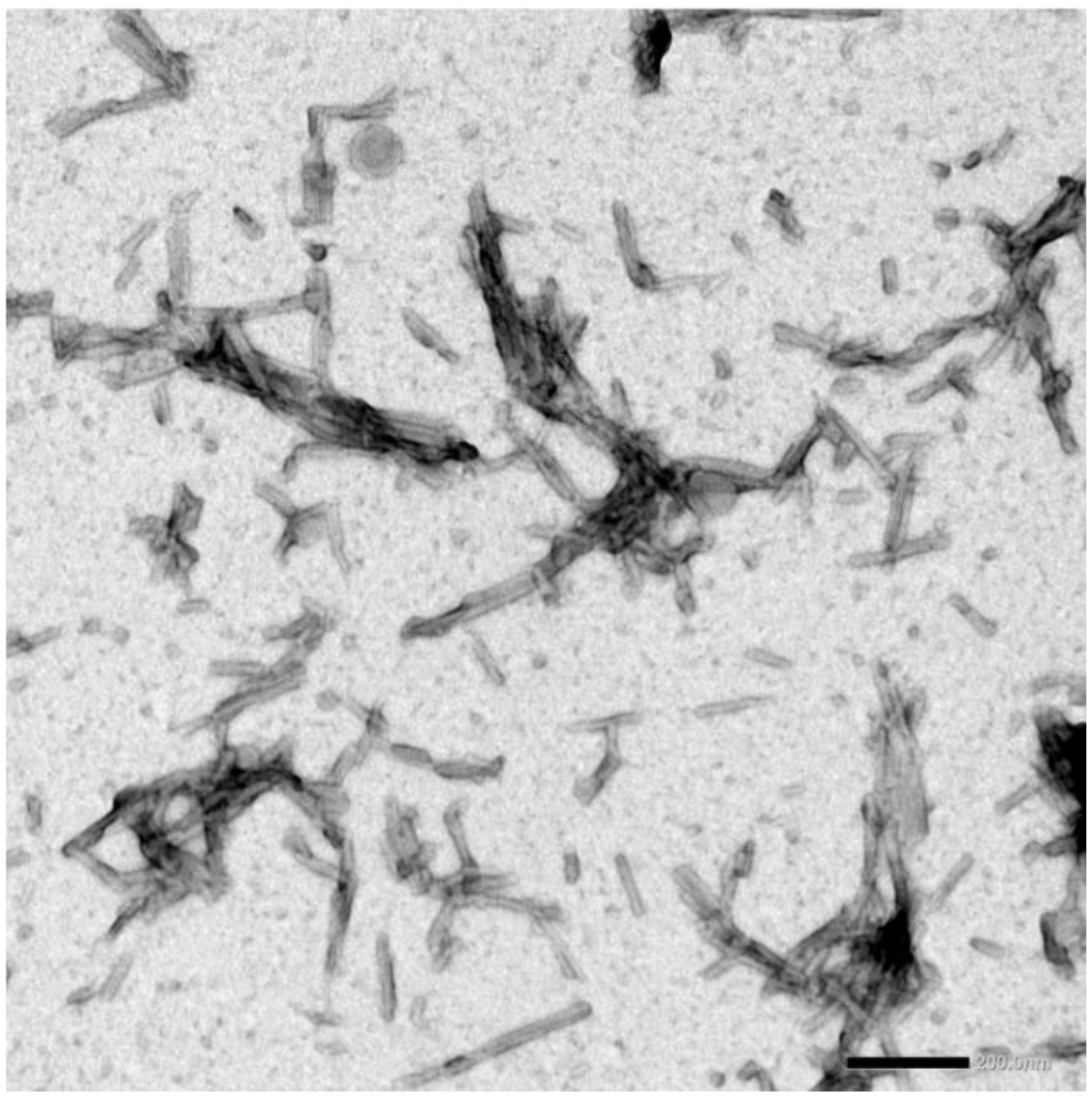

Supplementary Figure 1. Generation of α-synuclein PFFs. (A) Representative EM image of α-synuclein aggregates produced by shaking preparations for 7 days. Scale bar of 200 nm.

Supplementary Figure 2:

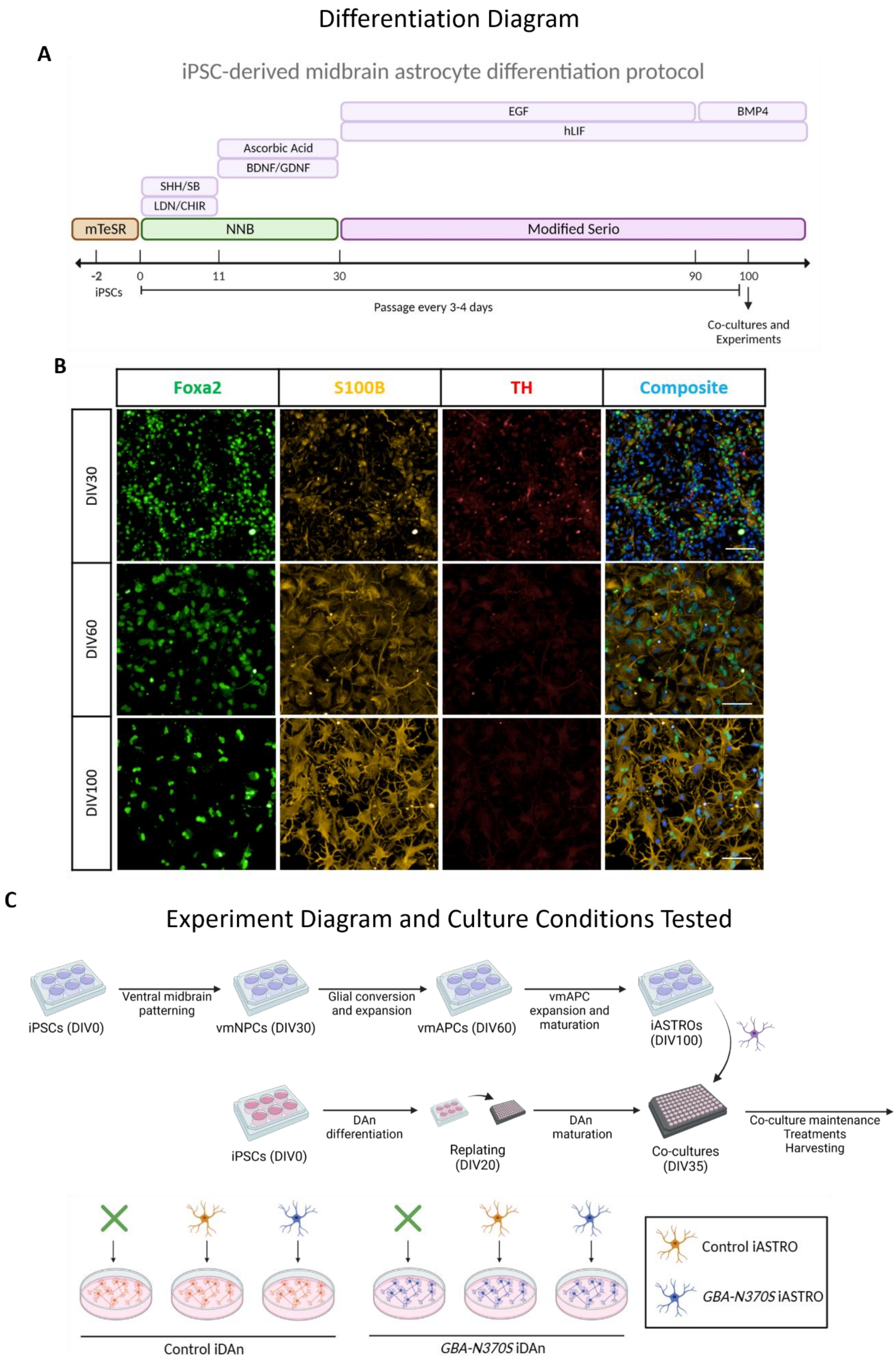

Supplementary Figure 2. Control and *GBA-N370S* iASTROs express mature astrocytic markers and present functional maturity. (A) Schematic diagram of iPSC-derived midbrain astrocyte differentiation protocol. (B) Representative images of midbrain dopaminergic neuron (Foxa2, TH) and astrocytic (S100B) markers expression at DIV30, DIV60 and DIV100 of differentiation protocol, stained for Foxa2 (green), S100B (yellow), TH (red) and DAPI nuclear staining (blue). (C) Schematic diagram of iPSC-derived ventral midbrain astrocyte differentiation protocol and iDAn-iASTRO co-culture.

Supplementary Figure 3:

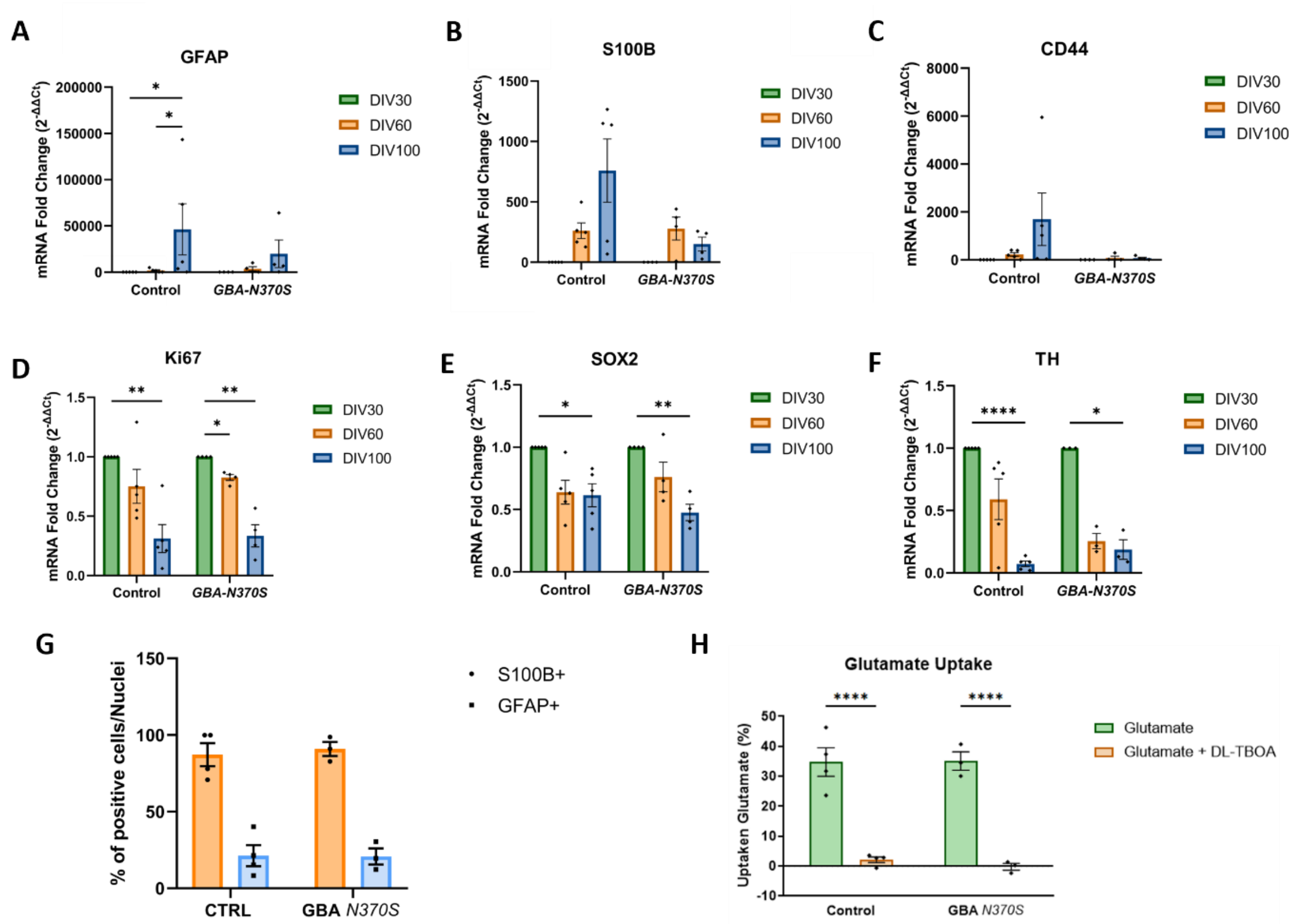

Supplementary Figure 3. Control and *GBA-N370S* iASTROs express mature astrocytic markers and present functional maturity II. (A-F) Relative gene expression analysis by RT-qPCR of several astrocytic, midbrain and proliferative markers at DIV30, DIV60 and DIV100 of differentiation protocol. (G) Quantification of the percentage of S100+ and GFAP+ cells over total nuclei at DIV100 of iASTRO differentiation. (H) Quantification of the percentage of the glutamate taken up in control and GBA-N370S iASTROs incubated with 100 μM glutamate, untreated or treated with 100 μM of the EAAT inhibitor DL-TBOA. Each data point represents 1 iASTRO line averaged across differentiations. N = 6 control and 4

GBA-N370S lines, 2-5 differentiations. The statistical analysis performed was two-way ANOVA with Fisher's LSD test. *p<0.05, **p<0.01, ***p<0.001, ****p<0.0001.

Supplementary Figure 4:

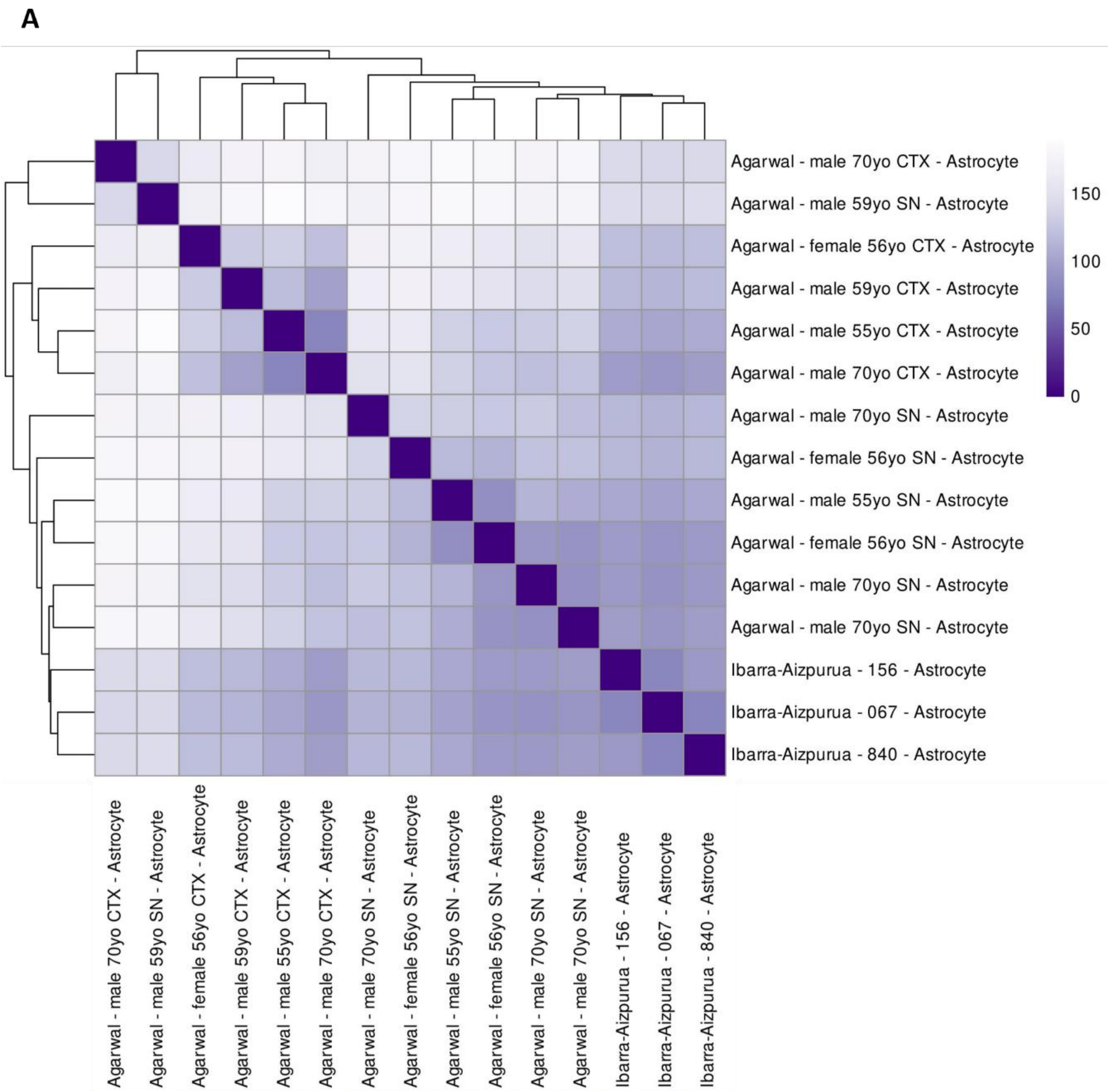

Supplementary Figure 4. Transcriptomic similarity between iASTROs and post-mortem human astrocytes.

(A) Heatmap of sample-to-sample Euclidean distances computed from batch-corrected, rlog-transformed RNAseq counts using the 1,000 most variable genes. Hierarchical clustering was performed using Euclidean distance. To statistically evaluate clustering, a permutation-based multivariate analysis of variance (PERMANOVA; 10,000 permutations) was performed on the distance matrix. Tissue identity [iASTRO, substantia nigra (SN), cortex (CTX)] significantly explained transcriptomic variation ($R^2 = 0.2140$, $p = 0.0413$). Homogeneity of group dispersion was confirmed

using a multivariate analogue of Levene's test for homogeneity of variances (p = 0.2255). For directional comparison, the mean Euclidean distance between each iASTRO sample and SN or CTX astrocyte pseudobulk samples was computed. iASTROs exhibited reduced mean distance to SN astrocytes compared to CTX astrocytes (paired t-test, one-sided, p = 0.0017, mean difference = -7.3547), supporting midbrain regional similarity.

Supplementary Figure 5:

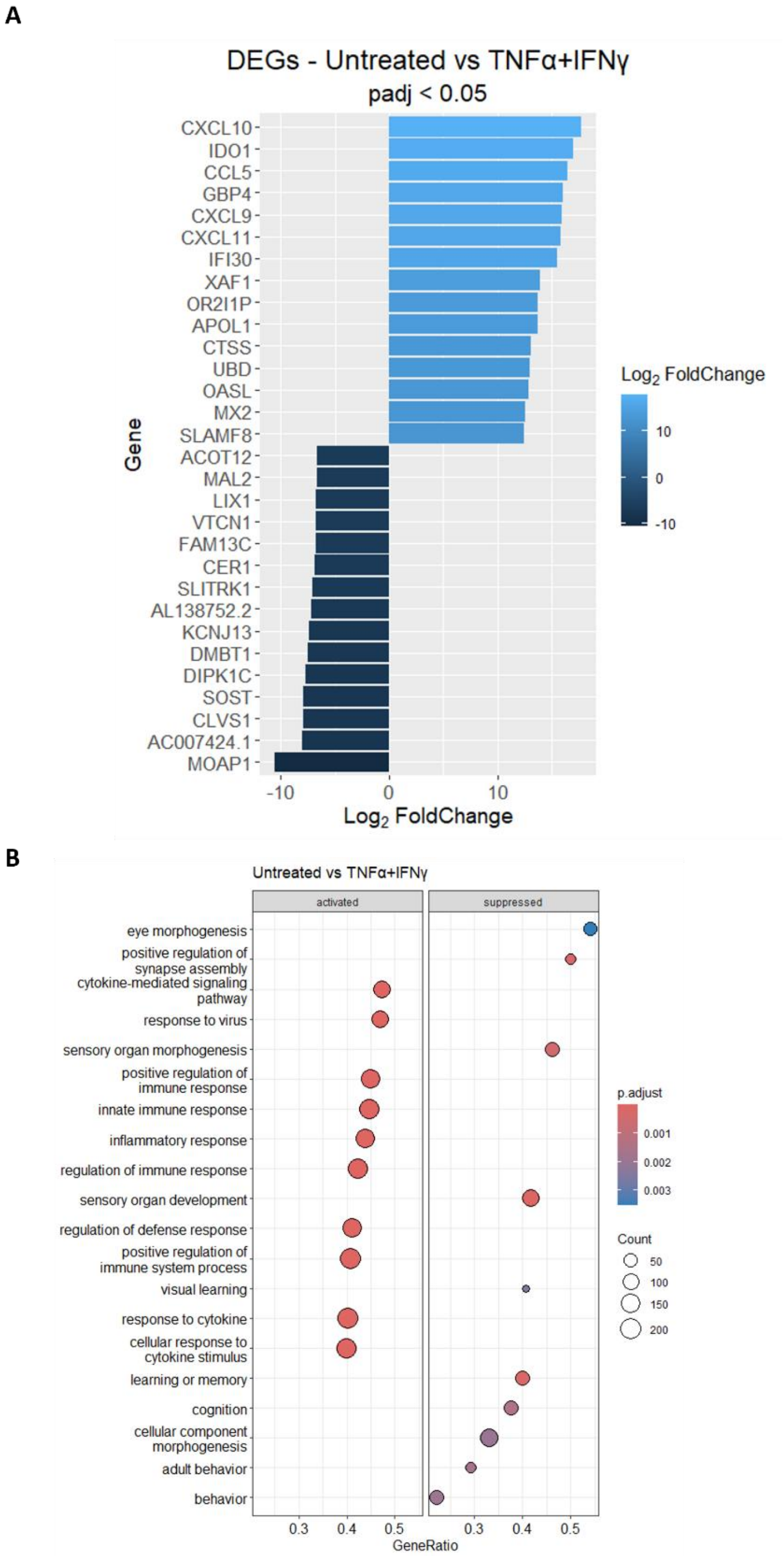

Supplementary 5. RNAseq exploration reveals wide-spread effects due to TNFα and IFNγ treatment in reactive astrocytes. Analysis of (A) the top 15 and bottom 15 differentially expressed genes and (B) the top 10 activated and top 10 suppressed gene ontology biological process terms in control iASTROs treated for 24h with 30 ng/mL TNF + 50 ng/mL IFNγ, compared to untreated iASTROs.

Supplementary Figure 6:

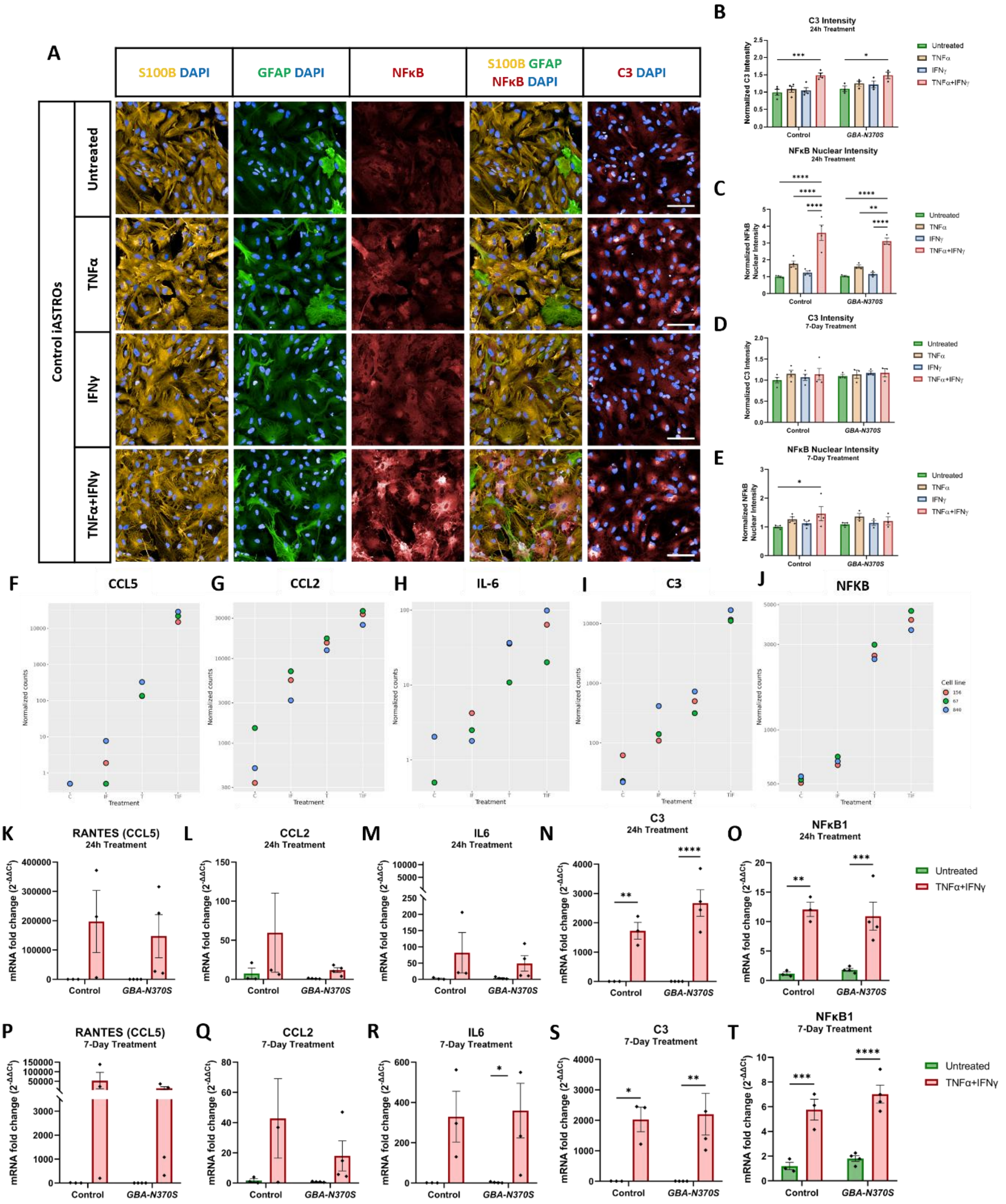

Supplementary Figure 6. Control and *GBA-N370S* iASTROs exhibit an inflammatory response after exposure to inflammatory cytokines. (A) Representative images of different astrocytic and inflammatory markers expression in control iASTROs, untreated or treated for 24 h with 30 ng/mL TNF, 50 ng/mL IFNγ or 30 ng/mL TNF + 50 ng/mL IFNγ, stained for S100B (yellow), GFAP (green), NFκB (red), C3 (red) and DAPI nuclear staining (blue). (B-E) Quantifications of C3 intensity and NFκB nuclear intensity in control iASTROs, untreated or treated for (B-C) 24 h or (D-E) 7 days with 30 ng/mL TNF, 50 ng/mL IFNγ or 30 ng/mL TNF + 50 ng/mL IFNγ. (F-J) RNAseq analysis of the normalized counts of *CCL5* (*RANTES*), *CCL2, IL-6, C3* and *NFκB* in control iASTROs untreated, or treated for 24h with 50 ng/mL IFNγ (IF), 30 ng/mL TNF (T) or 30 ng/mL TNF + 50 ng/mL IFNγ (TIF). Each dot color represents a different control iASTRO line. (K-T) Relative gene expression analysis by RT-qPCR of *CCL5* (*RANTES*), *CCL2*, *IL-6*, *C3* and *NFκB* in control and *GBA-N370S* iASTROs, untreated or treated for (K-O) 24 h or (P-T) 7 days with 30 ng/mL TNF + 50 ng/mL IFNγ. Each data point represents 1 iASTRO line averaged across differentiations. N = 3 control and 3 *GBA-N370S* lines, 3 differentiations. Mean ± SEM. The statistical analysis performed was two-way ANOVA with Dunnett's multiple comparison test to compare all treatment conditions to the untreated condition in each genotype, and two-way ANOVA with Šídák's multiple comparison test to compare both genotypes in each treatment condition. *$p<0.05$, **$p<0.01$, ***$p<0.001$.

Supplementary Figure 7:

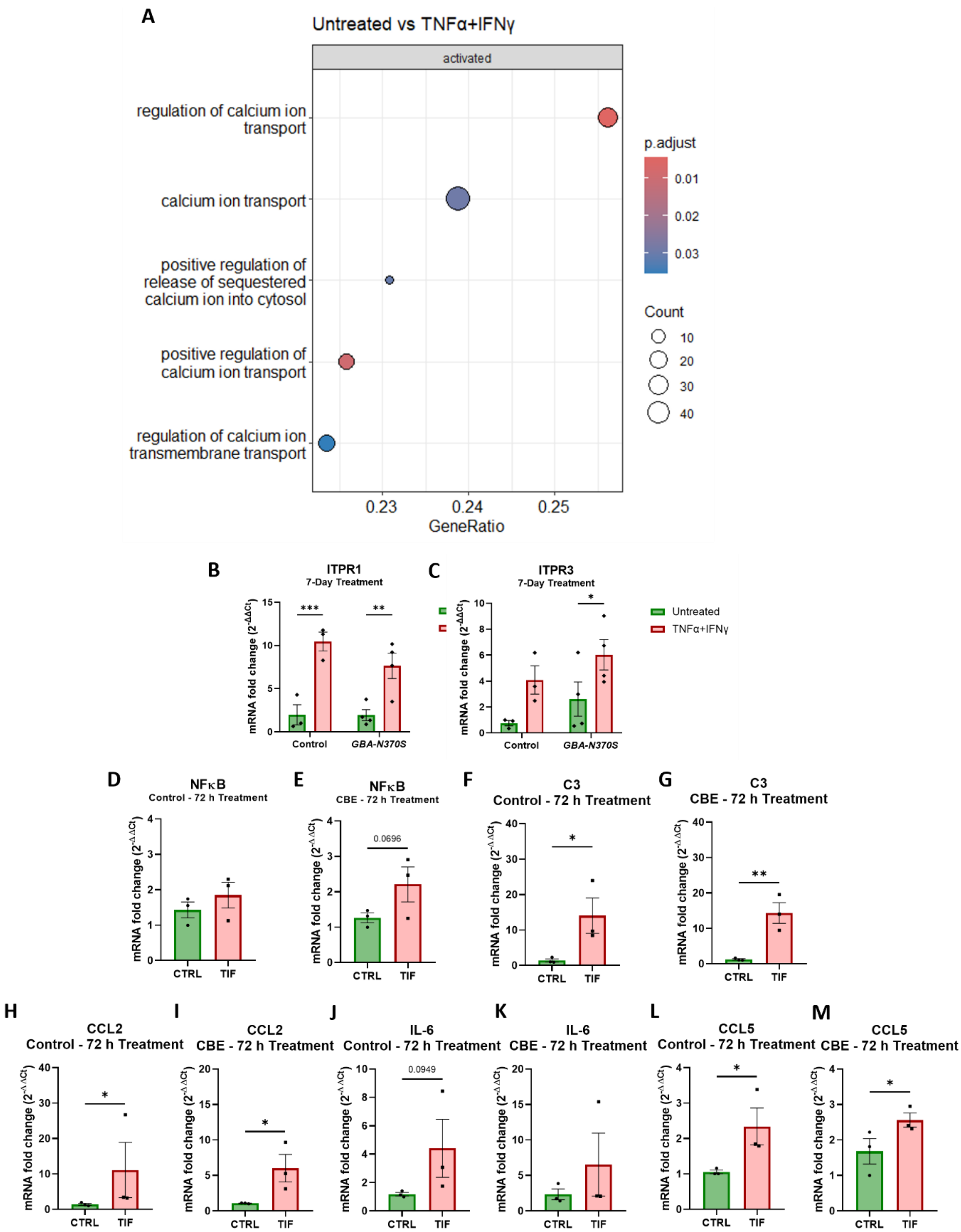

Supplementary Figure 7. RNAseq analysis reveals perturbations in calcium homeostasis and transport pathways in iASTROs after treatment with TNFα+IFNγ. (A) Gene ontology biological process terms related to calcium transport and homeostasis activated in iASTROs treated for 24h with

30 ng/mL TNFα + 50 ng/mL IFNγ compared to untreated iASTROs. (B-M) Relative gene expression analysis by RT-qPCR of specified genes in (B-C) control and *GBA-N370S* iASTROs, untreated or treated for 7 days with 30 ng/mL TNF + 50 ng/mL IFNγ, and (D-M) primary cultures treated with CBE, and/or 30 ng/mL TNF + 50 ng/mL IFNγ for 72 h. Each data point represents an independent primary culture or 1 iASTRO line averaged across differentiations. N = 3 control and 4 *GBA-N370S* lines, 1-4 differentiations. Mean ± SEM. The statistical analysis performed was two-way ANOVA with Tukey's multiple comparison test. *$p<0.05$, **$p<0.01$, ***$p<0.001$.

Supplementary Figure 8:

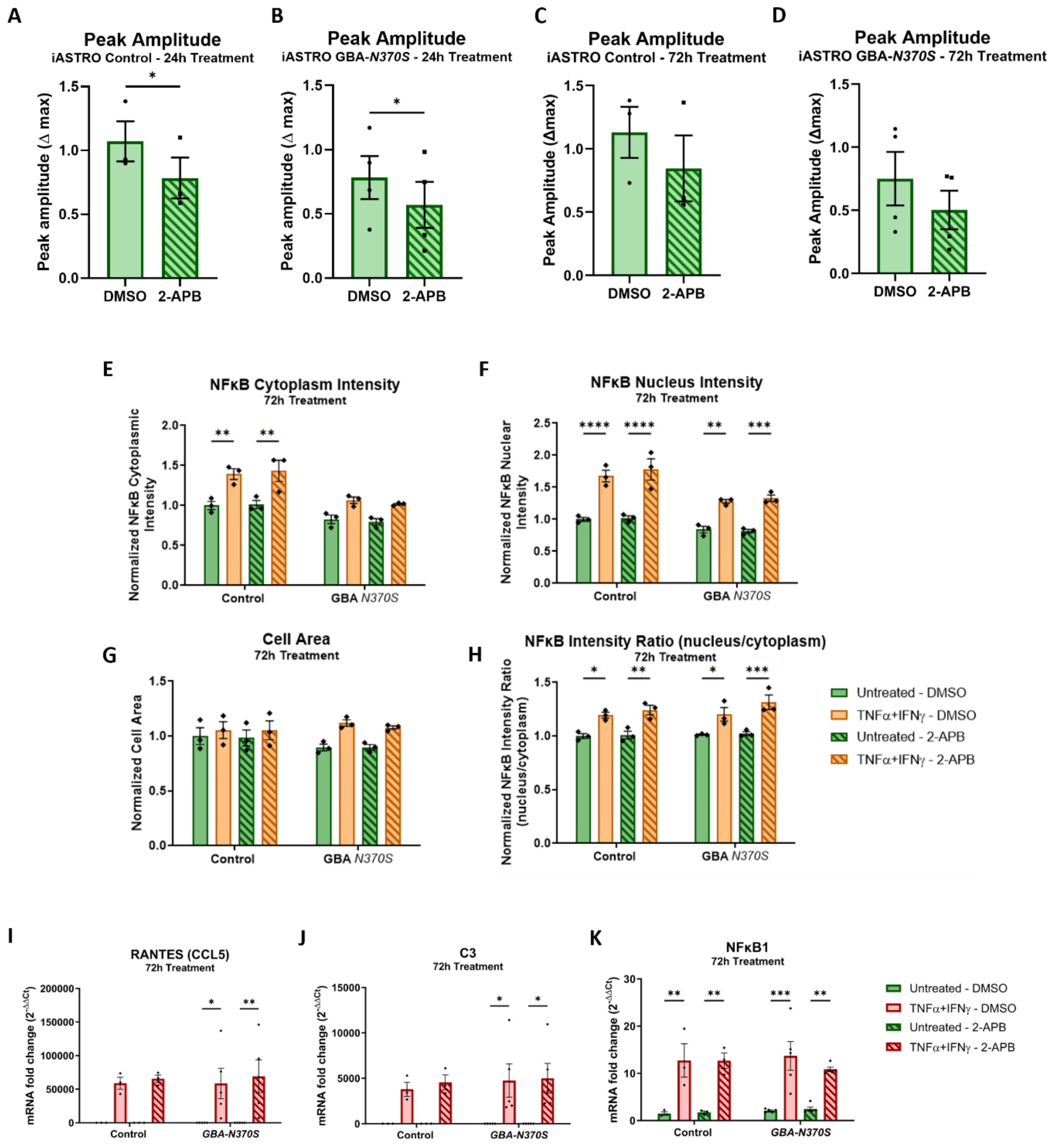

Supplementary Figure 8. Calcium release from the ER is reduced with the ITPR blocker 2-APB, but this does not alter overall astrocyte reactivity. (A-D) Quantifications of the peak amplitude of control and *GBA-N370S* iASTROs, untreated or treated for (A-B) 24 h or (C-D) 72 h with 25 µM 2-APB. Each data represents 1 line averaged across differentiations. N = 3 control and 4 *GBA-N370S* lines, 3 differentiations. Mean ± SEM. The statistical analysis performed was unpaired t-test with Welch's correction. *$p<0.05$ (E-H) Quantification of NFκB cytoplasm, nucleus, cell are and nucleus/cytoplasm ratio intensity, in control and *GBA-N370S* iASTROs, untreated or treated for 72 h with 25 µM 2-APB, 30 ng/mL TNF + 50 ng/mL IFNγ, or 25 µM 2-APB+30 ng/mL TNF + 50 ng/mL IFNγ. (I-K) Relative gene

expression analysis by RT-qPCR of RANTES/CCL5, C3 and NFκB in control and *GBA-N370S* iASTROs, untreated or treated for 72 h with 25 μM 2-APB, 30 ng/mL TNF + 50 ng/mL IFNγ, or 25 μM 2-APB+30 ng/mL TNFα + 50 ng/mL IFNγ. Each data point represents 1 iASTRO line averaged across differentiations. N = 3 control and 3 *GBA- N370S* lines, 3 differentiations. Mean ± SEM. The statistical analysis performed was two-way ANOVA with Tukey's multiple comparison test. *p<0.05, **p<0.01, ***p<0.001, ****p<0.0001.

Supplementary Figure 9:

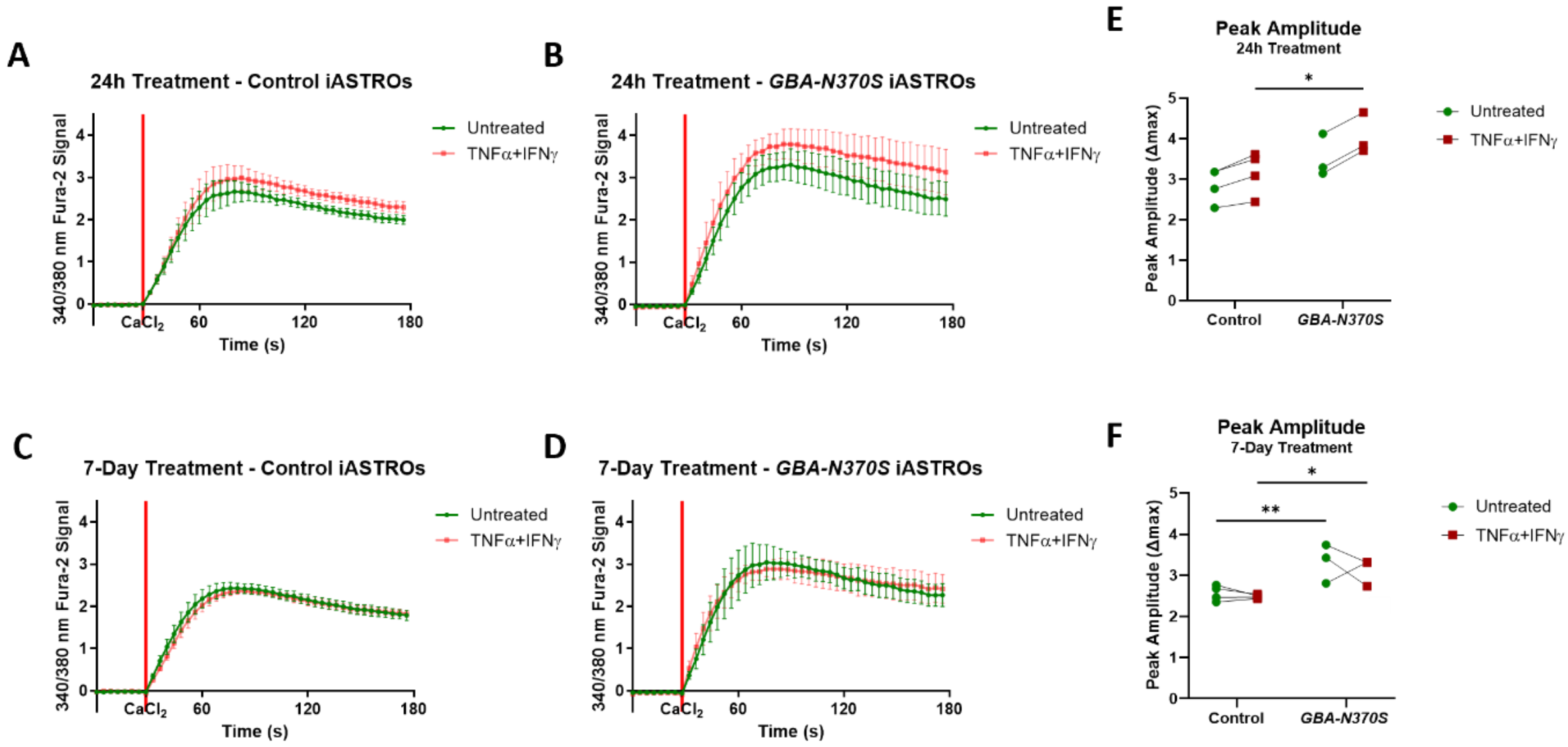

Supplementary Figure 9. Store-operated calcium entry (SOCE) is dysregulated in reactive astrocytes. (A-D) Traces showing the average of Fura-2 ratio in control and *GBA-N370S* iASTROs, untreated or treated for (A-B) 24 h and (C-D) 7 days with 30 ng/mL TNF + 50 ng/mL IFNγ. (E-F) Quantifications of the peak amplitude of the traces shown in (A-D). Each data point represents one line averaged across differentiations. N = 4 control and 3 *GBA-N370S* lines, 1-3 differentiations. Mean ± SEM. The statistical analysis performed was two-way ANOVA with Tukey's multiple comparison test. *p<0.05, **p<0.01.

Supplementary Figure 10:

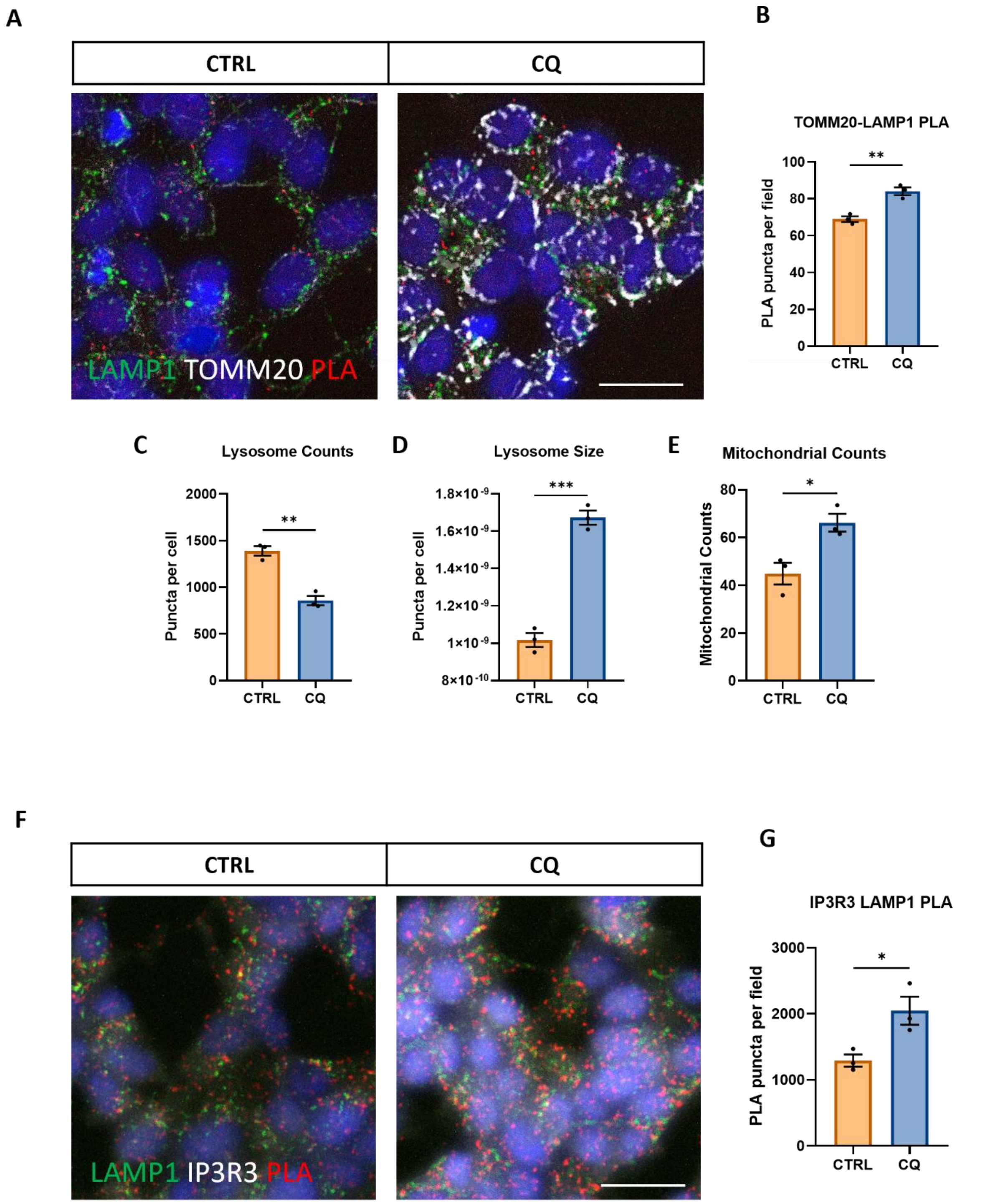

Supplementary Figure 10. Organellar interactions are disrupted by chloroquine in SH-SY5Y cells. (A) Representative images of LAMP1, TOMM2 and LAMP1-TOMM20 PLA signal in SH-SY5Y cells, untreated or treated for 24 h with 20 μM chloroquine (CQ), stained for LAMP1 (green), TOMM20 (grey), PLA (red) and DAPI nuclear staining (blue). (B) Quantification of TOMM20-LAMP1 PLA puncta

per field. (C-E) Quantifications of lysosomal counts and size, and of mitochondrial counts, in SH-SY5Y cells, untreated or treated for 24 h with 20 μM chloroquine. (F) Representative images of LAMP1, IP3R3 and IP3R3-LAMP1 PLA signal in SH-SY5Y cells, untreated or treated for 24 h with 20 μM chloroquine, stained for LAMP1 (green), IP3R3 (grey), PLA (red) and DAPI nuclear staining (blue). (G) Quantification of IP3R3-LAMP1 PLA puncta per field. Each data point represents one biological replicate. N = 3 biological replicates for control and CQ-treated SH-SY5Y cells. The statistical analysis performed was unpaired t test. *$p<0.05$, **$p<0.01$, ***$p<0.001$.

Supplementary Figure 11:

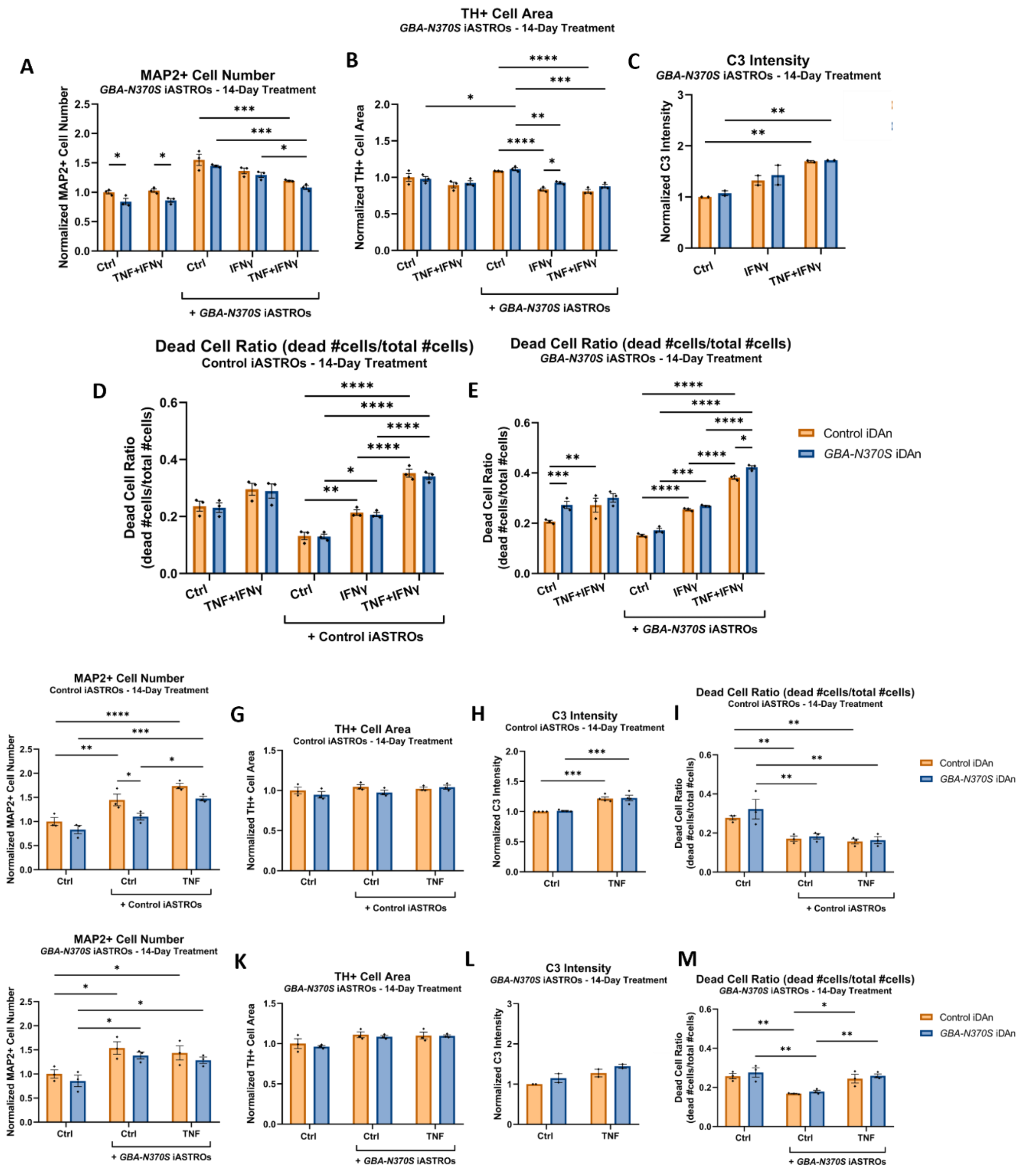

Supplementary Figure 11. Reduced neurosupportive effect by *GBA-N370S* iASTROs treated with TNF-α and IFN-γ. (A-C) Quantification of the number of MAP2+ cells, the cell area of TH+ cells, and the astrocytic cytoplasmic C3 intensity in iASTROs, in monoculture or in co-culture with *GBA-N370S* iASTROs, untreated or treated with IFNγ (50 ng/mL) or TNF (30 ng/mL) + IFNγ (50 ng/mL) for 14 days. (D-E) Quantification of the dead cell ratio, in monoculture or in co-culture with control and *GBA-N370S* iASTROs, untreated or treated with IFNγ (50 ng/mL) or TNF (30 ng/mL) + IFNγ (50 ng/mL) for 14 days.

(F-M) Quantification of the number of MAP2+ cells, the cell area of TH+ cells, the astrocytic cytoplasmic C3 intensity in iASTROs, and the dead cell ratio in monoculture or in co-culture with (F-I) control or (J-M) *GBA-N370S* iASTROs, untreated or treated with TNF (30 ng/mL) for 14 days. Each data point represents 1 iDAn line, averaged in each differentiation to all the conditions either in monoculture or co-cultured with 1-2 lines of control iASTROs, and then averaged across differentiations. For each differentiation, values were normalized to the average of untreated (Ctrl) control iDAn in monoculture. N = 3 control and 3 *GBA-N370S* lines for iDAn (3 differentiations), and N = 3 control lines for iASTROs (1- 2 differentiations per line). Mean ± SEM. The statistical analysis performed was two-way ANOVA with Tukey's multiple comparison test. $^*p<0.05$, $^{**}p<0.01$, $^{***}p<0.001$, $^{****}p<0.0001$. Not all statistically significant comparisons are shown in the graphs, due to the large number of comparisons with p values below 0.05.

Supplementary Figure 12:

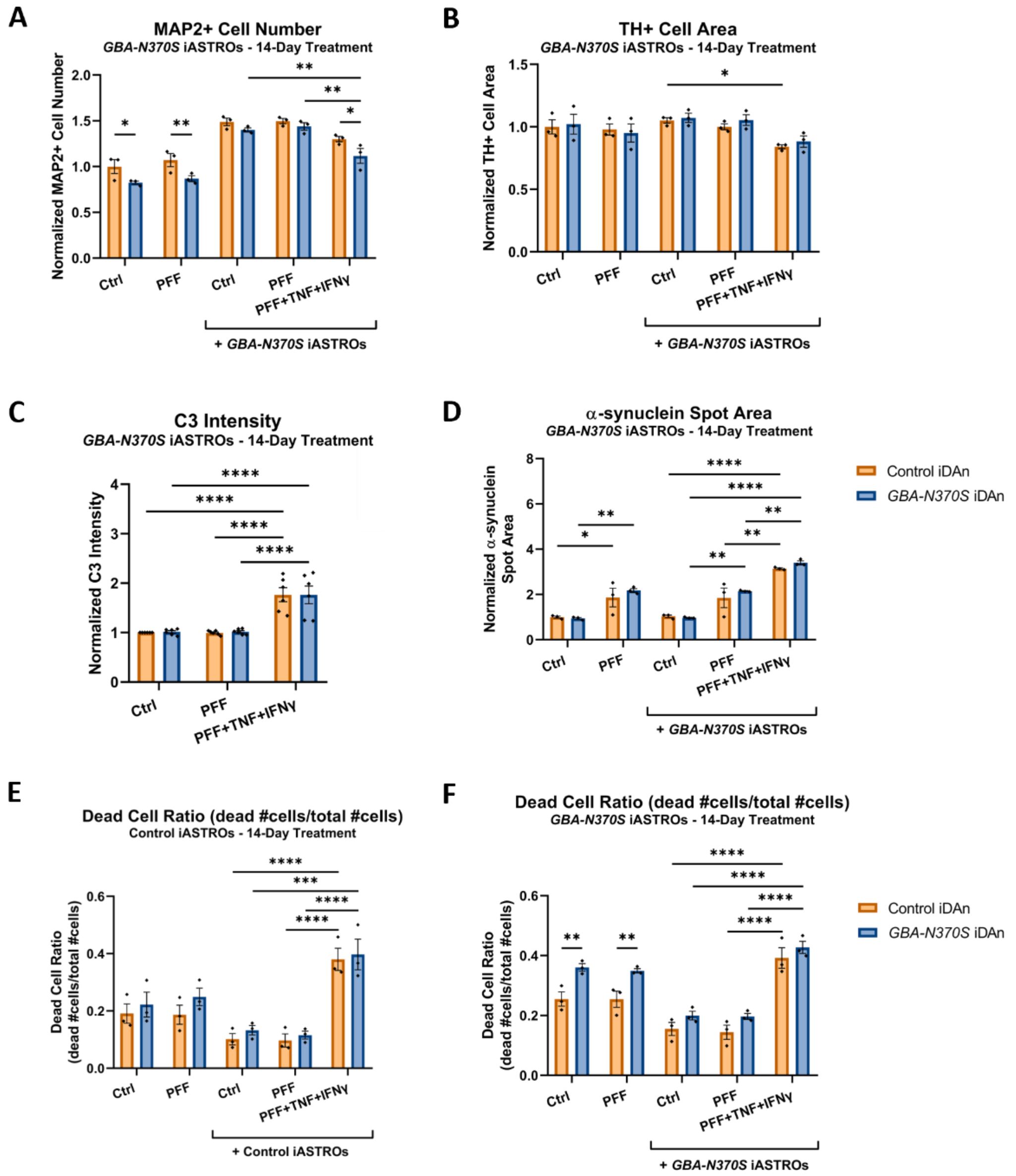

Supplementary Figure 12. Reduced neurosupportive effect by GBA-N370S iASTROs after TNF-α, IFN-γ and PFF treatment in iDAns-iASTRO co-cultures. (A-D) Quantification of the number of MAP2+ cells, the cell area of TH+ cells and the area of α-synuclein spots in control and *GBA-N370S* iDAn, and the astrocytic cytoplasmic C3 intensity in iASTROs, in monoculture or in co-culture with *GBA-N370S* iASTROs, untreated or treated with α-synuclein PFFs (10 μg/mL) or α-synuclein PFFs (10 μg/mL) + TNF (30 ng/mL) + IFNγ (50 ng/mL) for 14 days. (E-F) Quantification of the dead cell ratio, in monoculture or in co-culture with control and *GBA-N370S* iASTROs, untreated or treated with α-synuclein PFFs (10 μg/mL) or α-synuclein PFFs (10 μg/mL) + TNF (30 ng/mL) + IFNγ (50 ng/mL) for 14 days. Each data point represents 1 iDAn line averaged across differentiations. For each

differentiation, values were normalized to the average of untreated (Ctrl) control iDAn in monoculture. N = 3 control and 3 *GBA- N370S* lines for iDAn (4-5 differentiations), and N = 3 control lines for iASTROs (1-2 differentiations per line). Mean ± SEM. The statistical analysis performed was two-way ANOVA with Tukey's multiple comparison test. *p<0.05, **p<0.01, ***p<0.001, ****p<0.0001. Not all statistically significant comparisons are shown in the graphs, due to the large number of comparisons with p values below 0.05.

Supplementary Table 1:

**Comparison**	**DEGs**	**Log_2FoldChange>0**	**Log_2FoldChange<0**	**Log_2FoldChange >1.5**	**Log_2FoldChange<-1.5**
**Control vs TNF**	3,804	1,936	1,868	582	434
**Control vs Liddelow Factors**	4,793	2,445	2,348	756	601
**Control vs IFNγ**	4,818	2,461	2,357	744	593
**Control vs TNF+ IFNγ**	8,280	4,235	4,045	1,675	1,411
**Control vs α-synuclein PFFs**	17	6	11	5	2
**Control vs TNF+ IFNγ+PFFs**	8,446	4,282	4,164	1,683	1,540
**TNF vs Liddelow Factors**	258	183	75	88	19
**TNF vs IFNγ**	4,450	2,378	2,072	711	645

**TNF vs TNF+IFNγ**	6,685	3,610	3,075	1,312	843
**Liddelow Factors vs IFNγ**	4,946	2,602	2,344	749	760
**Liddelow Factors vs TNF+IFNγ**	6,857	3,680	3,177	1,273	923
**IFNγ vs TNF+IFNγ**	4,960	2,709	2,251	900	494
**PFFs vs TNF+IFNγ+PFFs**	8,271	4,204	4,067	1,685	1,487
**PFFs vs TNF+IFNγ**	8,137	4,193	3,944	1,659	1,3497
**TNF+IFNγ vs TNF+IFNγ+PFFs**	7	1	6	0	5

Supplementary Table 1. Upregulated and downregulated DEGs upon pro-inflammatory stimulation.

Supplementary Table 2:

Comparison	Activated GO:BP Terms	Suppresed GO:BP Terms
**Control vs TNF**	559	93
**Control vs Liddelow Factors**	623	71
**Control vs IFNγ**	437	51
**Control vs TNF+ IFNγ**	680	90
**Control vs α-synuclein PFFs**	2	0
**Control vs TNF+ IFNγ+PFFs**	729	31
**TNF vs Liddelow Factors**	0	0
**TNF vs IFNγ**	51	22
**TNF vs TNF+IFNγ**	368	115
**Liddelow Factors vs IFNγ**	32	68
**Liddelow Factors vs TNF+IFNγ**	274	125
**IFNγ vs TNF+IFNγ**	392	98
**PFFs vs TNF+IFNγ+PFFs**	732	34
**PFFs vs TNF+IFNγ**	714	72
**TNF+IFNγ vs TNF+IFNγ+PFFs**	0	0

Supplementary Table 2. Activated and suppressed Gene Ontology: Biological Process (GO:BP) terms upon pro-inflammatory stimulation.
